# *In vitro* and *in vivo* inhibition of breast cancer cell growth by targeting the Hedgehog/GLI pathway with SMO (GDC-0449) or GLI (GANT-61) inhibitors

**DOI:** 10.18632/oncotarget.7062

**Published:** 2016-01-28

**Authors:** Monica Benvenuto, Laura Masuelli, Enrico De Smaele, Massimo Fantini, Rosanna Mattera, Danilo Cucchi, Elena Bonanno, Enrica Di Stefano, Giovanni Vanni Frajese, Augusto Orlandi, Isabella Screpanti, Alberto Gulino, Andrea Modesti, Roberto Bei

**Affiliations:** ^1^ Department of Clinical Sciences and Translational Medicine, University of Rome “Tor Vergata”, Rome, Italy; ^2^ Department of Experimental Medicine, University of Rome “Sapienza”, Rome, Italy; ^3^ Department of Molecular Medicine, University of Rome “Sapienza”, Rome, Italy; ^4^ Department of Biomedicine and Prevention, University of Rome “Tor Vergata”, Rome, Italy; ^5^ Department of Physical Education, Human Sciences and Health, University of Rome “Foro Italico”, Rome, Italy

**Keywords:** breast cancer, Hedgehog/GLI pathway, inhibitors, tumor growth, tumor model

## Abstract

Aberrant Hedgehog (Hh)/glioma-associated oncogene (GLI) signaling has been implicated in cancer progression. Here, we analyzed GLI1, *Sonic Hedgehog* (Shh) and NF-κB expression in 51 breast cancer (ductal carcinoma) tissues using immunohistochemistry. We found a positive correlation between nuclear GLI1 expression and tumor grade in ductal carcinoma cases. Cytoplasmic Shh staining significantly correlated with a lower tumor grade. Next, the *in vitro* effects of two Hh signaling pathway inhibitors on breast cancer cell lines were evaluated using the *Smoothened* (SMO) antagonist GDC-0449 and the direct GLI1 inhibitor GANT-61. GDC-0449 and GANT-61 exhibited the following effects: a) inhibited breast cancer cell survival; b) induced apoptosis; c) inhibited Hh pathway activity by decreasing the mRNA expression levels of GLI1 and Ptch and inhibiting the nuclear translocation of GLI1; d) increased/decreased EGFR and ErbB2 protein expression, reduced p21-Ras and ERK1/ERK2 MAPK activities and inhibited AKT activation; and e) decreased the nuclear translocation of NF-κB. However, GANT-61 exerted these effects more effectively than GDC-0449. The *in vivo* antitumor activities of GDC-0449 and GANT-61 were analyzed in BALB/*c* mice that were subcutaneously inoculated with mouse breast cancer (TUBO) cells. GDC-0449 and GANT-61 suppressed tumor growth of TUBO cells in BALB/*c* mice to different extents. These findings suggest that targeting the Hh pathway using antagonists that act downstream of SMO is a more efficient strategy than using antagonists that act upstream of SMO for interrupting Hh signaling in breast cancer.

## INTRODUCTION

The Hedgehog (Hh)/glioma-associated oncogene (GLI) pathway is a complex signaling cascade that performs crucial functions in vertebrate embryogenesis and adult tissue homeostasis [1]. Three Hh homologs have been identified in vertebrates: *Sonic Hedgehog* (Shh), *Indian Hedgehog* (Ihh) and *Desert Hedgehog* (Dhh) [2, 3].

Hh ligands initiate “canonical” Hh signaling by binding to a 12-span transmembrane protein receptor termed *Patched-1* (Ptch), which is located at the base of a non-motile structure that protrudes from the cell surface, known as the “primary cilium” [3, 4].

In the absence of an Hh ligand, Ptch represses signal transduction by inhibiting the 7-span transmembrane protein *Smoothened* (SMO) from entering the cilium. Upon ligand binding, SMO enters the cilium and transduces the Hh signal, activating the cytoplasmic GLI family of zinc-finger transcription factors and promoting their translocation to the nucleus. Three GLI proteins are involved in vertebrate Hh signaling; GLI1 and GLI2 stimulate but GLI3 antagonizes the function of Shh-GLI1/2 [3, 4].

GLI activation induces the transcription of Hh target gene products, including ubiquitous genes such as GLI1, Ptch1 and Hh-interacting protein (Hhip) and cell type-specific genes such as Cyclin D, Myc, Bmi1, Bcl-2, vascular endothelial growth factor (VEGF), angiopoietins and SNAIL, depending on the cell type [3, 5]. In addition, Hh signaling down-regulates E-cadherin [3, 5].

GLI protein activation is controlled at different levels *via* phosphorylation or acetylation by inhibitors such as Suppressor of Fused (SuFu), REN/KCTD11/KCASH1, protein kinase A (PKA), and glycogen synthase kinase 3b (GSK3b) and activators such as Dyrk1, Ras and AKT [6-10].

Aberrant Hh signaling, which can be achieved by mutational inactivation of Ptch, aberrant expression of its ligand, constitutive activation of SMO or gene amplification of GLI-associated transcription factors, has been implicated in the initiation and/or maintenance of different cancer types, including basal cell carcinoma (BCC), gastrointestinal, lung, and brain tumors and rhabdomyosarcoma [3]. In addition, dysregulation of Hh signaling can be involved in the development and progression of breast cancer [11]. Mutations in Hh pathway genes have been identified at a low frequency in breast cancer cases, although no function of these mutations in breast cancer has been shown [12-15]. Conversely, several studies reported the overexpression of an Hh ligand, often Shh, and the Hh transcriptional targets GLI1 and Ptch1, thus activating the Hh pathway, in breast cancer [11, 16-19]. Shh expression was up-regulated in early-stage breast carcinoma, suggesting that the up-regulation of Shh may be an early event in breast carcinogenesis [19]. Furthermore, the positive correlation of NF-κB expression with Shh up-regulation suggests that NF-κB controls Shh expression in breast cancer [19]. Indeed, accumulating evidence has indicated that the Hh/GLI signaling cascade contributes to malignant transformation *via* cross-talk with ErbB receptors and NF-κB [4, 20, 21].

Targeting the Hh pathway could be a promising therapy for several types of tumors. More than 50 compounds have been identified to inhibit Hh signaling in cancer [22]. In particular, GDC-0449 (Vismodegib/Erivedge^TM^), an SMO antagonist, has entered clinical trials and was approved in January 2012 by the FDA for the treatment of adults with locally advanced or metastatic BCC who cannot be treated with surgery or radiation [23, 24]. Another promising therapeutic agent is GANT-61, which directly binds to the transcription factor GLI [25]. The efficacy of blocking the Hh pathway using GANT-61 is under investigation in many preclinical studies [25].

In this study, we assessed the expression levels of GLI1, Shh and NF-κB in 51 ductal breast carcinoma specimens by immunohistochemical analysis and their correlations with clinico-pathological variables. Furthermore, we explored the *in vitro* effects of GDC-0449 and GANT-61 on cell proliferation, cell cycle regulation and Hh, ErbB receptors and pro-survival signaling pathways in human (MDA-MB-231, MDA-MB-453, MDA-MB-468, T47-D, MCF-7, BT-474, and SK-BR-3) and mouse (TUBO) breast cancer cell lines. In addition, we evaluated the *in vivo* antitumor activities of these two inhibitors in BALB/*c* mice that were transplanted with TUBO cells. Our results demonstrate the aberrant expression of Hh signaling pathway members in breast carcinoma and the *in vitro* and *in vivo* anti-cancer activities of two Hh pathway inhibitors.

## RESULTS

### GLI1, Shh and NF-κB expression in human breast carcinoma specimens

To assess the expression of GLI1, Shh and NF-κB in breast cancer tissues, immunohistochemical analysis was performed using specific antibodies. Staining for GLI1, Shh and NF-κB was analyzed by employing a tissue microarray composed of 51 ductal breast carcinoma specimens (G1: *n* = 10; G2: *n* = 31; and G3: *n* = 10). The expression of GLI1, Shh and NF-κB correlated with clinico-pathological variables including histological type, tumor grade, tumor size, lymph node metastasis, and EGFR, ErbB2, estrogen receptor (ER) and progesterone receptor (PR) expression (Table 1).

**Table 1:** Expression of GLI1, Shh and NF-κB in ductal breast carcinoma specimens and their correlation with clinico-pathological variables

		Number of Specimens	GLI1 Nuclear Pos (%)*	Shh		NF-κB Pos (%)*
				Cytoplasmic Pos (%)*	Nuclear Pos (%)*	
Histological Type	Invasive Ductal	51	33 (41±19)	30 (97±13)	5 (86±31)	20 (100)
T Classification	T1 T2 T3 T4	18 29 1 3	11 (41±22) 19 (40±19) 1 (60) 2 (35±7)	13 (100) 15 (93±18) 0 2(100)	2 (100) 3 (77±40) 0 0	11^e^(100) 9^e^(100) 0^e^ 0^e^
Grading of Invasive Ductal	G1 G2 G3	10 31 10	3^a^(15±5)^b^ 23^a^(45±19)^b^ 7^a^(40±14)^b^	4 (100)^d^ 19 (100)^d^ 7 (86±24)^d^	1 (100) 4 (83±35) 0	4 (100) 15 (100) 1 (100)
Lymph node involvement	Negative Positive	25 26	18 (41±22) 15 (41±17)	12 (100) 18 (94±16)	3 (77±40) 2 (100)	8 (100) 12 (100)
EGFR	Negative (0-1+) Positive (2-3+)	50 1	32 (40±19) 1 (70)	30 (97±13) 0	5 (86±31) 0	20 (100) 0
ErbB2	Negative (0-1+) Positive (2-3+)	43 8	26 (37±18)^c^ 7 (55±18)^c^	25 (96±14) 5 (100)	4 (100) 1 (30)	17 (100) 3 (100)
ER	Negative Positive	16 35	12 (42±19) 21 (40±20)	8 (88±23) 22 (100)	1 (100) 4 (83±35)	5 (100) 15 (100)
PR	Negative Positive	23 28	16 (44±20) 17 (43±19)	13 (96±14) 17 (97±12)	1 (100) 4 (83±35)	9 (100) 11 (100)

* mean nuclear staining (%)±standard deviation.

a Fisher's exact test: G2 *vs* G1 *p* = 0.022; G2+G3 *vs* G1 *p* = 0.023;

b Student's T test: G2 *vs* G1 *p* = 0.017; G3 *vs* G1 *p* = 0.020; G2+G3 *vs* G1 *p* = 0.012;

c Student's T test: ErbB2 positive *vs* negative *p* = 0.027;

d Student's T test: G2 *vs* G3 *p* = 0.014; G1+G2 *vs* G3 *p* = 0.007;

e Fisher's exact test: T1 *vs* T2+T3+T4 *p* = 0.034.

Nuclear expression of GLI1 was observed in 33/51 (65%) carcinoma samples, including 3/10 (30%), 23/31 (74%) and 7/10 (70%) G1, G2 and G3 tumors, respectively. The nuclear GLI1 staining was heterogeneous, ranging from 10% to 80% of all cells, with a mean percentage of 41%. The number of carcinoma specimens and the percentage of tumor cells displaying nuclear GLI1 expression were significantly associated with the tumor grade (Table 1). Nuclear GLI1 expression was observed in 26/43 (60%) ErbB2-negative tumor samples and 7/8 (88%) ErbB2-positive tumor samples, and the mean percentage of cells with nuclear GLI1 staining was 37 and 55%, respectively. The percentage of cells displaying nuclear GLI1 staining positively correlated with ErbB2 positivity (*p* = 0.027) in the carcinoma samples. However, nuclear GLI1 staining did not correlate with the expression of the other analyzed receptors, and no correlation was found between GLI1 expression and lymph node involvement or tumor size.

Cytoplasmic Shh expression was observed in 30/51 (59%) carcinoma samples (4/10 (40%) of G1, 19/31 (61%) of G2 and 7/10 (70%) of G3 samples). The mean percentage of cells displaying cytoplasmic Shh staining was 97%. The percentage cells displaying cytoplasmic Shh staining correlated with the tumor grade. In addition, we observed cytoplasmic Shh expression in 25/43 (58%) ErbB2-negative and 5/8 (63%) ErbB2-positive tumors, in 13/23 (57%) PR-negative and 17/28 (61%) PR-positive tumors and in 8/16 (50%) ER-negative and 22/35 (63%) ER-positive tumors. However, no correlation between cytoplasmic Shh staining and expression of these receptors was found.

Notably, nuclear Shh staining was found in 5/51 (10%) carcinoma samples. However, no correlation between nuclear Shh staining and any clinico-pathological variable was detected.

**Figure 1:**
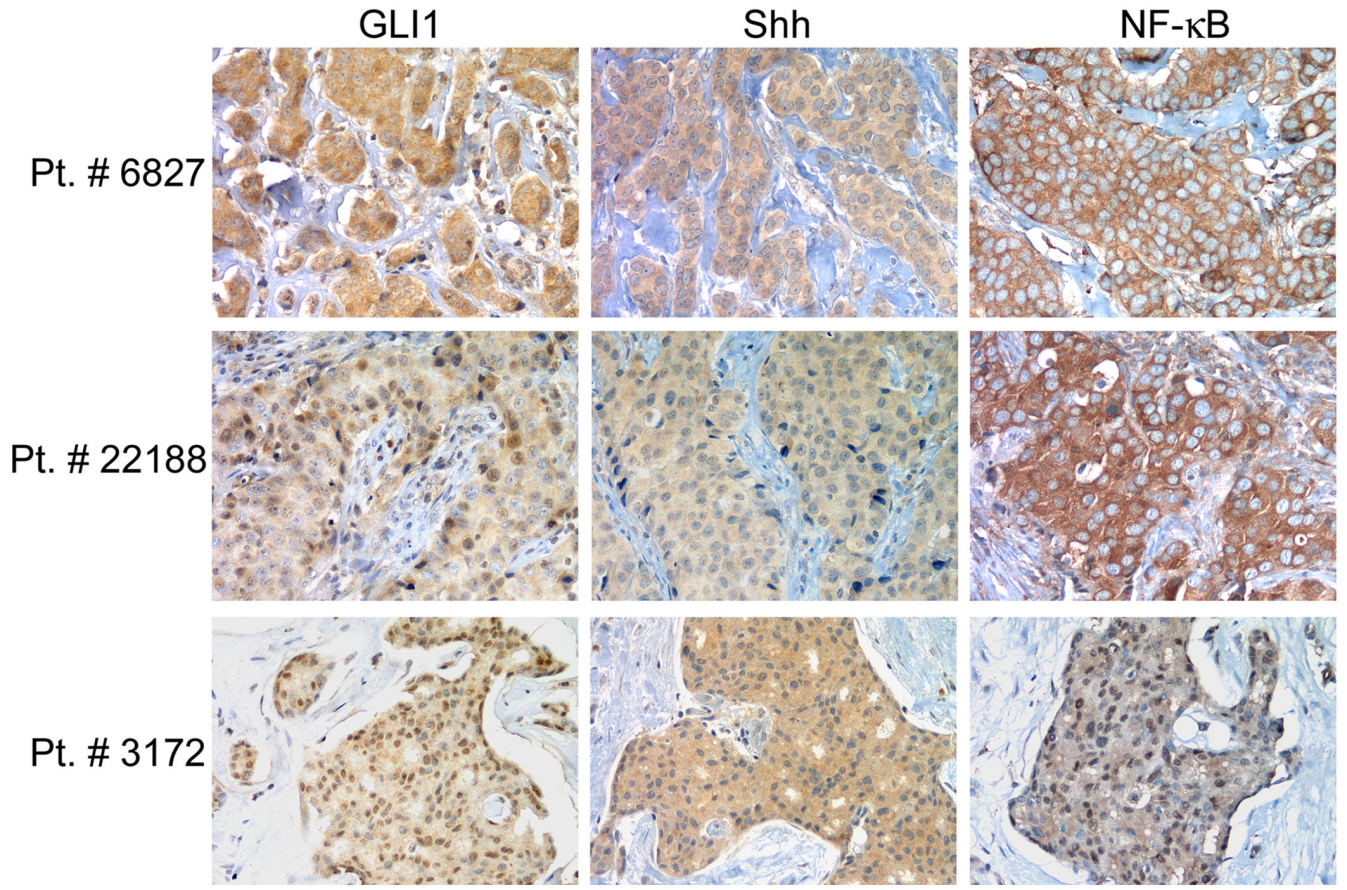
GLI1, Shh and NF-κB expression in human breast carcinoma specimens Representative immunohistochemical staining of samples from 3 patients with ductal carcinoma. The immunoreactivity of the samples was visualized by immunoperoxidase staining using specific antibodies as described in the “Materials and methods” section. Original magnification, 200x.

NF-κB expression was observed in 20/51 (39%) carcinoma specimens (4/10 (40%) for G1; 15/31 (48%) G2; and 1/10 (10%) G3 samples). In addition, we found NF-κB expression in 17/43 (40%) ErbB2-negative and 3/8 (38%) ErbB2-positive tumors; in 9/23 (39%) PR-negative and 11/28 (39%) PR-positive tumors; and in 5/16 (31%) ER-negative and 15/35 (43%) ER-positive tumors. NF-κB expression in the carcinoma specimens positively correlated with smaller tumor size (*p* = 0.034). Representative immunostaining results are shown in Figure 1.

### Inhibition of breast cancer cell survival by GDC-0449 and GANT-61

The aberrant expression of Hh signaling pathway members in breast carcinoma samples prompted us to evaluate the *in vitro* effects of two Hh signaling pathway inhibitors on breast cancer cell lines. GDC-0449 is an SMO antagonist, and GANT-61 directly inhibits GLI.

The survival of human (MCF-7, T47-D, MDA-MB-231, MDA-MB-468, MDA-MB-453, BT-474, and SK-BR-3) and mouse (TUBO) breast cancer cell lines was evaluated by the sulforhodamine B (SRB) assay after exposing the cells to increasing doses of GDC-0449 (3-12 μM) or the vehicle control (DMSO) for 48, 72, 96 hours or 6 days. The same assay was performed after exposing the cells to increasing doses of GANT-61 (1-20 μM) or the vehicle control (DMSO) for 48 and 72 hours. Experiments were also performed using TUBO cells to establish whether this mouse cell line was susceptible to the *in vitro* antitumor activity of these inhibitors and would thus be suitable for transplantation into BALB/*c* to generate tumors for the purpose of determining the *in vivo* antitumor effects of these two inhibitors.

**Figure 2:**
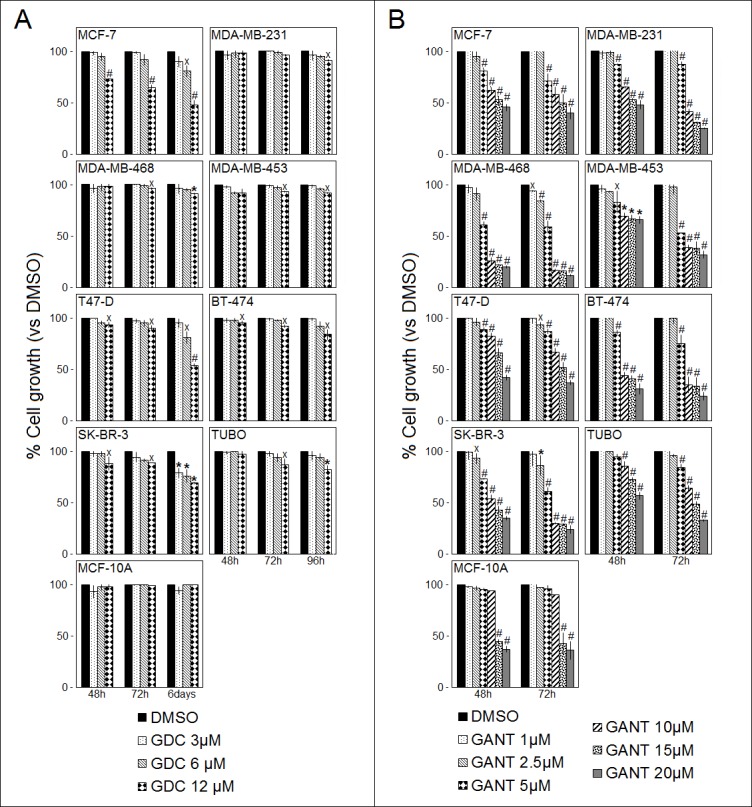
Effects of GDC-0449 (GDC) and GANT-61 (GANT) on the survival of breast cancer cells The survival of human (MCF-7, T47-D, MDA-MB-231, MDA-MB-468, MDA-MB-453, BT-474, and SK-BR-3) and mouse (TUBO) breast cancer cell lines and of human MCF-10A mammary epithelial cells were assessed by the SRB assay after 48, 72, 96 hours or 6 days of treatment with DMSO or GDC-0449 (Panel **A.**) or after 48 and 72 hours of treatment with DMSO or GANT-61 (Panel **B.**). The percentage of surviving cells treated with a compound or the vehicle control was calculated by normalizing the O.D. value to that of the untreated control cultures. The results are expressed as the means±SD of three independent experiments performed in triplicate (^˟^*p* ≤ 0.05, *p ≤ 0.01, ^#^*p* ≤ 0.001 compared with the cultures treated with DMSO).

GDC-0449 decreased cell survival in all cell lines, although only at the highest dose (12 μM) and after 72 and 96 hours or 6 days of treatment. This effect was particularly evident in MCF-7, T47-D, SK-BR-3 and BT-474 cells, in which GDC-0449 significantly reduced cell survival at 12 μM after 48 hours. In addition, GDC-0449 reduced MCF-7 and T47-D cell survival at 6 μM and SK-BR-3 cell survival at 6 and 3 μM after 6 days of treatment. The mean results of three independent experiments are reported in Figure 2, panel A.

The effect of GANT-61 on cell proliferation was dose- and time-dependent and was significant compared with the vehicle control at doses from 5 to 20 μM in 7 out of the 8 examined cell lines after 48 hours and in all cell lines after 72 hours of treatment. GANT-61 significantly reduced cell survival at all doses tested after 72 hours only in MDA-MB-468 cells. GANT-61 significantly decreased cell survival in SK-BR-3 cells (after 48 and 72 hours) and T47-D cells (after 72 hours) when applied at the 2.5 μM dose. The mean results of three independent experiments are reported in Figure 2, panel B.

To evaluate the non-specific toxic effects of these drugs, the survival of human MCF-10A mammary epithelial cells, which are a non-tumorigenic epithelial cell line derived from mammary glands, was evaluated upon treatment with each compound (Figure 2, panel A and B). Notably, GDC-0449 did not alter MCF-10A cell survival. In addition, GANT-61 significantly reduced MCF-10A cell survival only at the highest doses (20 and 15 μM), suggesting an *in vitro* toxic effect of this compound when applied at these high doses. No effects of GANT-61 on MCF-10A cells were observed at lower doses.

Overall, our results demonstrated that GANT-61 is more effective than GDC-0449 in reducing the survival of breast cancer cells.

### Effect of GDC-0449 and GANT-61 on cell cycle distribution in breast cancer cell lines

To evaluate the effect of GDC-0449 and GANT-61 on cell cycle distribution, FACS analysis of DNA content was performed on 8 breast cancer cell lines treated with increasing doses of GDC-0449 (3 to 25 μM) for 96 hours or 6 days or GANT-61 (5 to 20 μM) for 48 hours. DMSO was used as the vehicle.

**Figure 3:**
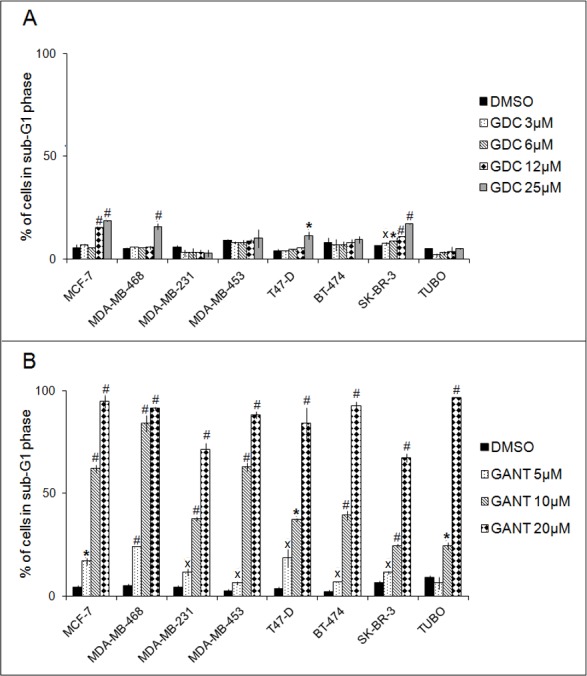
Effects of GDC-0449 (GDC) and GANT-61 (GANT) on the proportion of cells in sub-G1 phase of the cell cycle FACS analysis of DNA content was performed on breast cancer cells treated for 96 hours (MDA-MB-231, MDA-MB-453, BT-474, TUBO) or 6 days with DMSO or GDC-0449 (Panel **A**) or for 48 hours with DMSO or GANT-61 (Panel **B**). The results are expressed as the means±SD of three independent experiments performed in triplicate (^˟^*p* ≤ 0.05, *p ≤ 0.01, ^#^*p* ≤ 0.001 compared with the cultures treated with DMSO).

GDC-0449 treatment at the highest dose increased the percentage of MDA-MB-468 and T47-D cells in the sub-G1 phase and exerted the same effect on MCF-7 cells at 25-12 μM and on SK-BR-3 cells at all doses tested (Figure 3, Panel A). The increase in the percentage of cells in sub-G1 phase was associated with a decrease in the percentage of cells in G0/G1 phase in MDA-MB-468, T47-D, and SK-BR-3 cells and with a reduction in the percentage of cells in S and G2/M phases in MCF-7 cells. The mean results of three independent experiments are reported in Supplementary Table 1.

Treatment with GANT-61 dose-dependently increased the percentage of cells in sub-G1 phase in all cell lines. Pooling the data from these 8 cell lines, the percentage of cells in sub-G1 phase ranged from 2.00 to 8.98% in cultures treated with DMSO; from 6.31 to 23.91% in cultures treated with 5 μM GANT-61; from 24.28 to 84.12% in cultures treated with 10 μM GANT-61; and from 67.37 to 96.59% in cultures treated with 20 μM GANT-61 (Figure 3, Panel B). The increase in the sub-G1 fraction was associated with dose-dependent decreases in the percentage of cells in G0/G1, S or G2/M phases. The mean results of three independent experiments are reported in Supplementary Table 2.

In agreement with our above results, GANT-61 treatment appears to be more effective than GDC-0449 in increasing the percentage of cells in sub-G1 phase in all cell lines. Indeed, GANT-61 was effective after 48 hours, but GDC-0449 was effective only in 4 cell lines after 6 days of treatment.

### Effects of GDC-0449 and GANT-61 on p53, Bax, Bcl-2 and Poly(ADP-ribose) polymerase-1 (PARP-1)

To corroborate that the GDC-0449- and GANT-61-induced increase in the percentage of cells in sub-G1 phase was partially caused by the induction of apoptosis, PARP-1 cleavage and the Bax/Bcl-2 expression ratio were analyzed by Western blotting.

T47-D, MCF-7 and SK-BR-3 breast cancer cells were treated with 12 μM GDC-0449 or DMSO for 6 days. Alternatively, T47-D, MCF-7, SK-BR-3, MDA-MB-231, MDA-MB-468, MDA-MB-453 and BT-474 breast cancer cells were treated with 10 μM GANT-61 or DMSO for 48 hours. After incubation, the cells were lysed, resolved in 10% SDS-PAGE, transferred to nitrocellulose membranes and incubated with specific antibodies. The results varied according to the cell line examined, likely because different breast cancer cell lines have distinct phenotypes and genetic backgrounds (ER, PR, ErbB2, p53 and Ras expression statuses) [26-31] (Supplementary Table 3).

PARP-1, an enzyme that controls DNA repair, cell cycle progression and cell death, is cleaved by caspases during the apoptotic process. This cleavage can be detected by Western blotting using a specific antibody that recognizes both the full-length and C-terminally cleaved forms of PARP-1.

**Figure 4:**
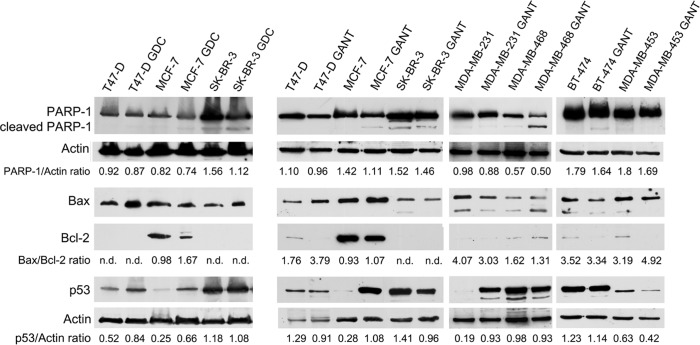
PARP-1 cleavage, the Bax/Bcl-2 expression ratio and the p53 expression level in breast cancer cell lines treated with GDC-0449 (GDC) or GANT-61 (GANT) Western blotting was performed on cells that were treated with GDC-0449 (12 μM) or DMSO for 6 days or with GANT-61 (10 μM) or DMSO for 48 hours. n.d. = not detected.

In GDC-0449-treated cells, although no increase in PARP-1 cleavage was observed, decreased PARP-1 expression was detected in SK-BR-3 cells (*p* = 0.02). In addition, the amount of pro-apoptotic Bax protein and the Bax/Bcl-2 ratio were increased following GDC-0449 treatment compared with DMSO treatment in all cell lines. Bcl-2 expression was not detected in T47-D and SK-BR-3 cells. The increased Bax/Bcl-2 expression ratio in MCF-7 cells and the increased Bax expression level in T47-D and SK-BR-3 cells might shift the cellular balance toward a pro-apoptotic state (Figure 4 and Supplementary Table 4).

Treatment with GANT-61 induced PARP-1 cleavage in MCF-7, MDA-MB-468 and BT-474 cells but not in the other cell lines. However, decreased PARP-1 expression in MCF-7 cells was observed after treatment with GANT-61 (*p* = 0.03). GANT-61 treatment increased the expression of the pro-apoptotic factor Bax and decreased the expression of Bcl-2 in MCF-7 (*p* = 0.03), T47-D (*p* < 0.001) and MDA-MB-453 (*p* = 0.006) cells. These events can shift the cellular balance towards a pro-apoptotic state. The expression of these apoptotic regulatory proteins was not significantly altered by GANT-61 treatment in the other cell lines (Figure 4 and Supplementary Table 4).

Next, to determine whether GDC-0449- or GANT-61-induced apoptosis requires p53, the expression of p53 was analyzed. p53 expression was significantly increased in GDC-0449-treated MCF-7 (*p* = 0.02) and T47-D (*p* = 0.04) cells, but remained unchanged in SK-BR-3 cells (Figure 4 and Supplementary Table 4).

GANT-61 treatment increased p53 expression in MCF-7 and MDA-MB-231 cells (*p* = 0.005 and *p* = 0.002, respectively); decreased p53 expression in T47-D (*p* = 0.02), SK-BR-3 (*p* = 0.01) and MDA-MB-453 (*p* = 0.04) cells; and did not change p53 expression in the remaining cell lines (Figure 4 and Supplementary Table 4).

### Effects of GDC-0449 and GANT-61 on the GLI1, GLI2 and Ptch mRNA levels and the nuclear translocation of GLI1

**Figure 5:**
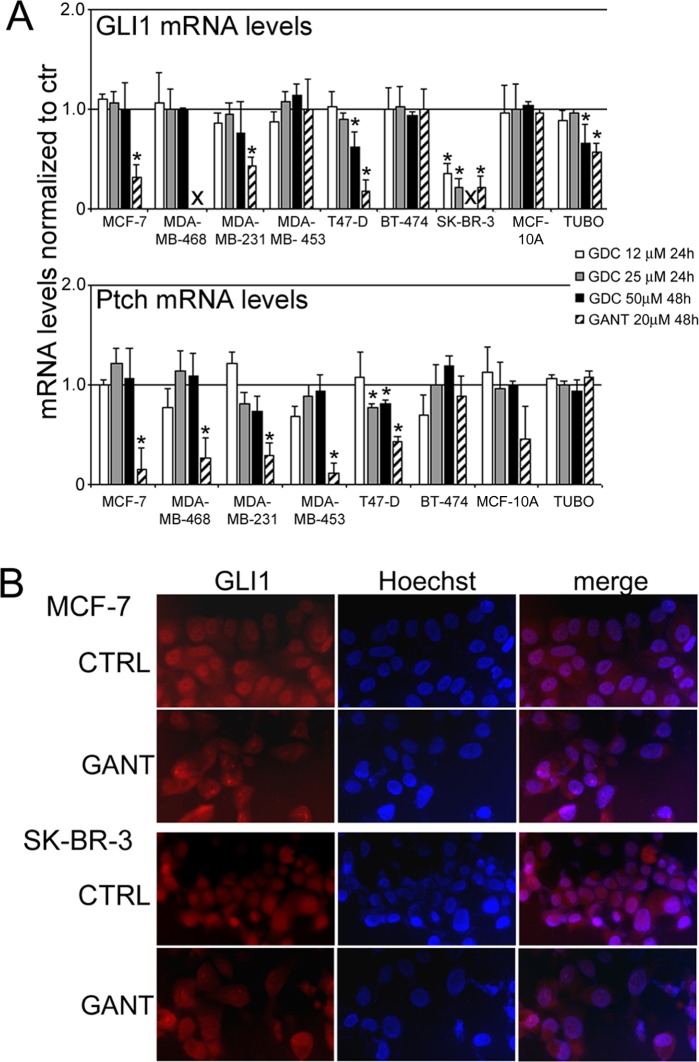
The GLI1 and Ptch mRNA levels and the nuclear translocation of GLI1 after treatment with GDC-0449 (GDC) or GANT-61 (GANT) in breast cancer cell lines Panel **A.** Real-time q-PCR was performed to assess the GLI1 and Ptch mRNA levels in RNA that was isolated from breast cancer cell lines that had been treated with GDC-0449 (12-25 μM for 24 hours and 50 μM for 48 hours), GANT-61 (20 μM for 48 hours) or DMSO as described in the “Materials and methods”. The GLI1 and Ptch mRNA expression levels were analyzed after normalization to the controls. The results are expressed as the mean values of three independent experiments (*p < 0.05 compared with the cultures treated with DMSO; X: the cells were dead). Panel **B.** Immunofluorescence analysis was performed on MCF-7 and SK-BR-3 breast cancer cells after treatment with GANT-61 (10 μM) or vehicle alone for 24 hours. After the treatment, the cells were fixed and incubated with an anti-GLI1 antibody, and after washing, the cells were labeled with an Alexa Fluor-594-conjugated goat anti-rabbit IgG antibody. The nuclei were counterstained with Hoechst. Original magnification, 400x.

To evaluate the effect of treatment with GDC-0449 or GANT-61 on the activity of the Hh pathway, real-time q-PCR was performed on RNA that was isolated from breast cancer cells treated with GDC-0449 (12-25 μM for 24 hours and 50 μM for 48 hours), GANT-61 (20 μM for 48 hours) or DMSO. After RNA isolation, cDNA synthesis was performed, and quantitative reverse transcription PCR (RT-PCR) analysis of the GLI1, GLI2 and Ptch mRNA expression levels was performed after normalization to the controls. The GLI1 and Ptch mRNA levels before and after cell treatment are reported in Figure 5, panel A.

Our results indicated that the GLI1 mRNA levels were greatly decreased after GDC-0449 treatment only in SK-BR-3 cells at 12-25 μM (*p* < 0.05); at 50 μM, GLI1 mRNA was not detectable. In T47-D and TUBO cells, the GLI1 mRNA levels were significantly decreased (*p* < 0.05) only at the highest dose of GDC-0449 (50 μM). No reduction in the GLI1 mRNA levels after GDC-0449 treatment was observed in the other cell lines. Conversely, GANT-61 treatment significantly decreased the GLI1 mRNA levels (*p* < 0.05) in SK-BR-3, T47-D, MCF-7, MDA-MB-231 and TUBO cells. In MDA-MB-468 cells, the GLI1 mRNA levels were not detectable even when lower doses of GANT-61 were applied.

The Ptch mRNA levels were slightly but significantly decreased after GDC-0449 treatment only in T47-D cells at high doses (25-50 μM). In the other cell lines, no reduction in the Ptch mRNA levels after GDC-0449 treatment was detected compared with the control treatment. Conversely, after GANT-61 treatment, a significant decrease (*p* < 0.05) in the Ptch mRNA levels in 5 of the 8 examined cell lines (T47-D, MCF-7, MDA-MB-231, MDA-MB-453 and MDA-MB-468) was observed. The Ptch mRNA levels were not detectable in SK-BR-3 cells (Figure 5, panel A). Accordingly, our results suggest that GANT-61 inhibits the Hh pathway more potently than GDC-0449. The distinct activities of GANT-61 and GDC-0449 on the GLI1 and Ptch mRNA levels in different cell lines might be due to variations in the basal activity of their promoters in these cells. Some of these cells possess different activated Hh components. GLI1 activation promotes its translocation to the nucleus to initiate the transcription of Hh target genes. To further corroborate the inhibitory effects of GANT-61 on the Hh pathway, immunofluorescence analysis was performed to detect GLI1 expression in MCF-7 and SK-BR-3 cells that were treated with 10 μM GANT-61 or DMSO for 24 hours. GANT-61 inhibited the translocation of GLI1 to the nucleus. Decreased nuclear expression of GLI1 was observed in MCF-7 and SK-BR-3 cells after GANT-61 treatment compared with the control treatment (Figure 5, panel B).

The GLI2 mRNA levels were very low and were unchanged in most the cell lines upon treatment with GDC-0449 or GANT-61. In addition, GLI2-overexpressing cell lines, except MDA-MB-231 and SK-BR-3, did not show a significant change in GLI2 mRNA levels following treatment. MDA-MB-231 and SK-BR-3 cells exhibited a significant reduction in the GLI2 mRNA levels only at the lowest dose of GDC-0449, thus indicating the presence of a compensatory mechanism that induces GLI2 transcription following strong inhibition of GLI target gene products (Supplementary Figure 1).

### Effects of GDC-0449 and GANT-61 on the expression and activation of ErbB receptors and pro-survival signaling pathway members

ErbB receptors are members of one of the primary signal transduction pathways implicated in cancer cell growth [32]. In addition, accumulating evidence has indicated that cross-talk between ErbB receptors and the Hh/GLI signaling pathway is involved in neoplastic transformation [20].

To evaluate whether GDC-0449 or GANT-61 alters EGFR/ErbB2 expression and ERK1/2 expression and activation, Western blotting analysis was performed after treating cells with GDC-0449 (12 μM) or DMSO for 6 days or with GANT-61 (10 μM) or DMSO for 48 h. The results varied according to the cell line used.

**Figure 6:**
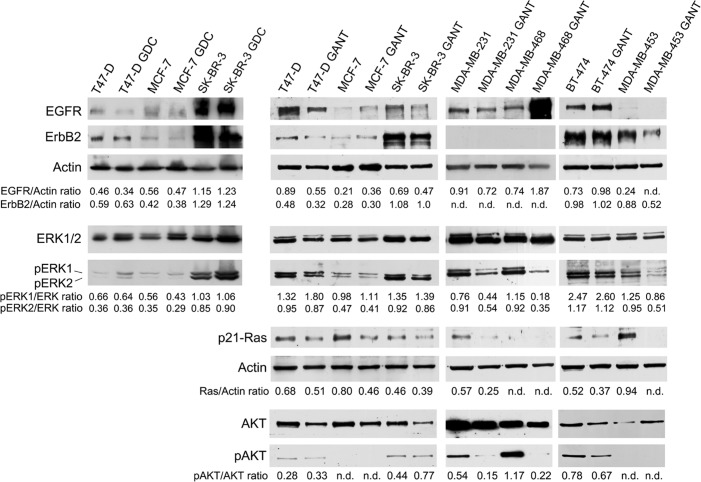
Effects of GDC-0449 and GANT-61 on the expression and activation of ErbB receptors and pro-survival signaling pathway members Western blotting was performed on cells that were treated with GDC-0449 (12 μM) or DMSO vehicle for 6 days or with GANT-61 (10 μM) or DMSO vehicle for 48 hours. The levels of p-ERK1/2 were compared with the total ERK1/2 protein level. The level of p-AKT was compared with the AKT protein level. n.d. = not detected.

GDC-0449 reduced EGFR expression in T47-D cells (*p* = 0.03) but did not change EGFR expression in MCF-7 and SK-BR-3 cells. GDC-0449 had no notable effect on the ErbB2 protein levels (Figure 6 and Supplementary Table 4).

In addition, GANT-61 slightly reduced EGFR expression in T47-D (*p* = 0.02), SK-BR-3 (*p* = 0.04) and MDA-MB-231 (*p* = 0.01) cells but increased EGFR expression in MCF-7 (*p* = 0.02), BT-474 (*p* = 0.004) and MDA-MB-468 (*p* = 0.006) cells. EGFR expression was not detectable after GANT-61 treatment in MDA-MB-453 cells, and this result suggested that this treatment reduced EGFR expression. GANT-61 decreased ErbB2 expression in T47-D (*p* = 0.008) and MDA-MB-453 (*p* = 0.005) cells but did not change its expression in MCF-7, SK-BR-3 and BT-474 cells; ErbB2 expression was not detectable in the other cell lines (Figure 6 and Supplementary Table 4).

We also investigated the effect of GDC-0449 or GANT-61 on the expression and phosphorylation of ERK1/ERK2 (ERK1/2), which are activated by ErbB receptors. The levels of phosphorylated ERK1 and ERK2 proteins were compared with the total ERK protein level. GDC-0449 did not alter the basal or phosphorylated ERK (p-ERK)1/2 protein expression levels in T47-D and SK-BR-3 cells, but GDC-0449 reduced the p-ERK1/2 levels in MCF-7 cells (*p* = 0.007 for p-ERK1; *p* = 0.03 for p-ERK2) (Figure 6 and Supplementary Table 4). The total ERK1/2 levels remained the same after GANT-61 treatment in all cell lines. GANT-61 significantly inhibited ERK1/2 phosphorylation in MDA-MB-231 (*p* = 0.049 for p-ERK1; *p* = 0.002 for p-ERK2), MDA-MB-468 (*p* = 0.04 for p-ERK1; *p* = 0.003 for p-ERK2) and MDA-MB-453 (*p* = 0.04 for p-ERK1; *p* = 0.009 for p-ERK2) cells (Figure 6 and Supplementary Table 4).

Ras is also activated by ErbB receptors [33]. Thus, we analyzed the effect of GANT-61 on Ras expression in breast cancer cell lines using an antibody that detects an epitope that is common to both activated and deactivated H-, K- and N-Ras p21. GANT-61 significantly inhibited p21-Ras expression in T47-D (*p* = 0.026), MCF-7 (*p* = 0.008), MDA-MB-231 (*p* = 0.006), BT-474 (*p* = 0.017), and MDA-MB-453 (*p* = 0.002) cells.

Furthermore, we evaluated whether GANT-61 treatment inhibits the protein expression of the pro-survival kinase AKT, which promotes tumor growth. GANT-61 significantly decreased the activated phosphorylated AKT (p-AKT) protein levels compared with the control in MDA-MB-231 (*p* = 0.004), MDA-MB-468 (*p* = 0.03) and BT-474 (*p* = 0.03) cells. p-AKT expression was not detectable in MCF-7 or MDA-MB-453 cells. GANT-61 treatment significantly increased the p-AKT/AKT ratio in SK-BR-3 cells compared with the control treatment (*p* = 0.02) (Figure 6 and Supplementary Table 4).

### Effects of GANT-61 on the nuclear translocation of NF-κB

**Figure 7:**
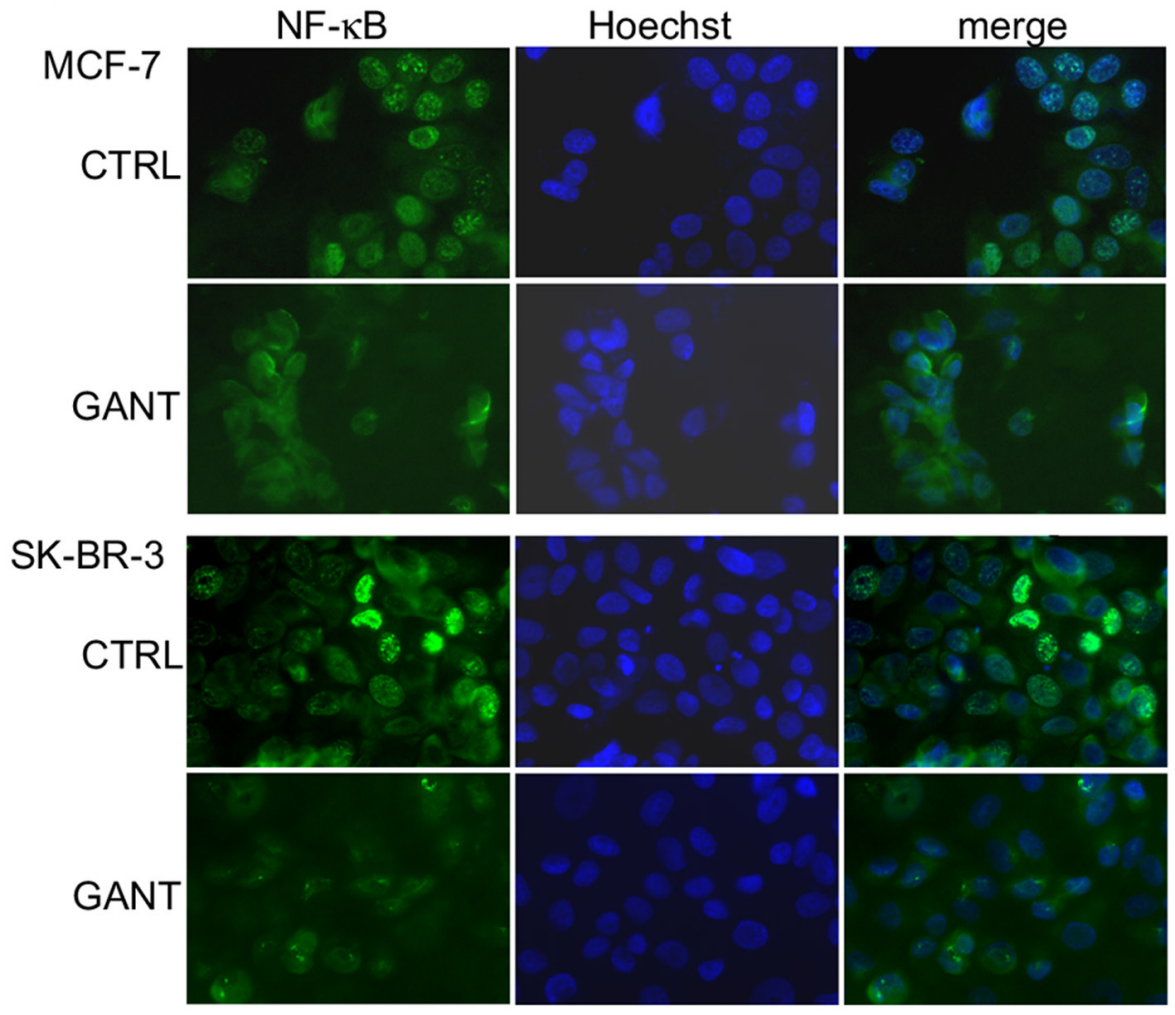
Inhibition of the nuclear translocation of NF-κB upon GANT-61 treatment Immunofluorescence analysis was performed on MCF-7 and SK-BR-3 breast cancer cells after treatment with GANT-61 (10 μM) or vehicle alone for 24 hours. After the treatment, the cells were fixed, incubated with an anti-NF-κB antibody, washed, and labeled with an Alexa Fluor-488-conjugated goat anti-mouse IgG antibody. The nuclei were counterstained with Hoechst. Original magnification, 400x.

Several studies suggest an interplay between the Hh and NF-κB signaling pathways in cancer cells. NF-κB activation induces its translocation from the cytoplasm to the nucleus. To evaluate whether GANT-61 treatment interferes with NF-κB nuclear translocation, immunofluorescence analysis was performed using MCF-7 and SK-BR-3 cells treated with 10 μM GANT-61 or DMSO for 24 h. GANT-61 treatment reduced the translocation of NF-κB to the nucleus of MCF-7 and SK-BR-3 cells compared to the control treatment (Figure 7).

### Effect of GANT-61 on ErbB2/*neu* and the pro-survival signaling protein AKT in murine TUBO cells

**Figure 8:**
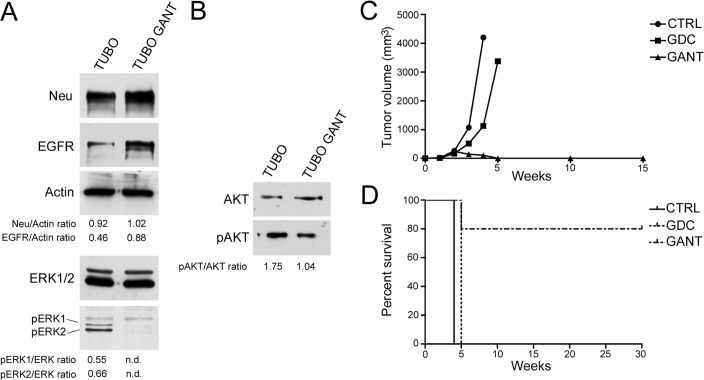
Effect of GANT-61 (GANT) on the protein expression of ErbB2/*neu*, EGFR, ERK1/2 and AKT in TUBO cells, and treatment with GDC-0449 (GDC) or GANT suppressed tumor growth in BALB/*c* mice that were subcutaneously inoculated with TUBO cells Western blotting was performed on murine mammary cancer (TUBO) cells that were treated with GANT-61 (10 μM) or DMSO vehicle for 48 hours. n.d. = not determined. Panel **A.** Effect of GANT-61 on *neu,* EGFR and ERK1/2 expression. The levels of p-ERK1/2 were compared with the total ERK1/2 protein levels. Panel **B.** Effect of GANT-61 on the AKT protein level. Panel **C.** Differences in mean tumor volumes between BALB/*c* mice treated with GDC-0449 (GDC), GANT-61 (GANT) or corn oil:ethanol (CTRL). Panel D: Differences in the mean survival duration of BALB/*c* mice treated with GDC-0449 (GDC), GANT-61 (GANT) or corn oil:ethanol (CTRL). The numbers of inoculated mice are reported in the “Materials and methods”.

To evaluate the effect of GANT-61 on ErbB2/*neu* and on the AKT protein in BALB-*neu*T mammary cancer cells (TUBO) overexpressing activated rat ErbB2/*neu,* the cells were treated with 10 μM GANT-61 for 48 h, followed by Western blotting analysis. Although an increase in EGFR expression was observed in GANT-61-treated cells relative to the control-treated cells (*p* = 0.04), GANT-61 actually decreased the p-ERK1-2/ERK ratio. Indeed, after GANT-61 treatment, ERK1 and ERK2 activation was abolished. However, no effect of GANT-61 on *neu* expression was observed (Figure 8, panel A). In addition, GANT-61 treatment decreased the p-AKT/AKT expression ratio compared with the control treatment (*p* = 0.01) (Figure 8, panel B).

Thus, our results suggest that GANT-61 exerts antitumor effects on TUBO cells by inhibiting the MAPK and AKT pathways.

### GDC-0449 and GANT-61 reduced tumor growth in BALB/*c* mice subcutaneously inoculated with TUBO cells

To evaluate the *in vivo* antitumor effects of GDC-0449 and GANT-61, groups of BALB/*c* female mice (5 mice per group) were subcutaneously inoculated with 1×10^6^ TUBO cells in the right flank. These mice were simultaneously treated *per os* with GDC-0449 or GANT-61 dissolved in corn oil:ethanol (4:1) or with the vehicle alone.

After 3 weeks of treatment, the mice treated with GDC-0449 or GANT-61 showed a significant decrease in the mean tumor volume compared with the control mice (513 compared with 1070 mm^3^, *p* = 0.0084 for GDC-0449; 142 compared with 1070 mm^3^, *p* = 0.0049 for GANT-61) (Figure 8, panel C). GDC-0449 increased the survival of BALB/*c* mice by one week compared with the control mice (*p* = 0.0009). After 4 weeks of treatment, the control mice were sacrificed because of the excessive size of their tumors. The GDC-0449-treated mice were sacrificed after 5 weeks of treatment (Figure 8, panel D). Conversely, GANT-61 administration counteracted TUBO cell growth in BALB/*c* mice. The mean tumor volume in GANT-61-treated mice was 238 mm^3^ two weeks after injection. In fact, 1 and 2 mice became tumor-free after 4 and 5 weeks of treatment, respectively (Figure 8, panel C). Overall, complete tumor regression was observed in 4/5 GANT-61-treated mice after 6 weeks of treatment (*p* = 0.0005 for GANT-61 compared with vehicle; *p* = 0.023 for GANT-61 compared with GDC-0449). These mice remained tumor-free for up to 30 weeks. Only 1 GANT-61-treated mouse died at 5 weeks after injection, although its tumor volume was small (500 mm^3^) (Figure 8, panel D).

Overall, our results indicated specific interference with TUBO tumor cell growth by Hh inhibitors, and the effect varied according to the compound. Regarding the survival of BALB/*c* mice upon treatment, the risk of TUBO tumor cell growth in the corn oil-treated group was 0.016 and 49.40 relative to the GDC-0449- and GANT-61-treated groups, respectively. In addition, the risk of TUBO tumor cell growth in the GDC-0449-treated group was 17.81 relative to the GANT-61-treated group (Table 2).

**Table 2:** Analysis of the survival of Balb/*c* mice after treatment with Hh inhibitors by the log-rank test (Mantel-Cox)

Variable	Contrast	Hazard ratio	95% hazard ratio confidence limits		*p* value
			lower	upper	
Treatment	GDC-0449 vs CTRL	0.016	0.001	0.19	0.0009
	GANT-61 vs CTRL	49.40	5.44	449	0.0005
	GANT-61 vs GDC-0449	17.81	1.47	216	0.0237

Mice were treated with GDC-0449 (GDC), GANT-61 (GANT) or corn oil:ethanol (CTRL).

## DISCUSSION

Aberrant Hh signaling has been implicated in the initiation and/or maintenance of different cancer types, including breast cancer [34]. Hh-induced carcinogenesis *via* constitutive Hh signaling activation is caused either by the mutation of Hh pathway components (ligand-independent) or by Hh overexpression (ligand-dependent). Mutations of Hh pathway genes have been identified at a low frequency in breast cancer, and no function of these mutations in breast cancer has been shown [12-15]. Alternatively, several studies reported overexpression of Hh ligands, often Shh, and of the Hh transcriptional targets GLI1 and Ptch1 and, thus, the activation of the Hh pathway in breast cancer [11, 16, 17].

Accordingly, we assessed the expression of Hh pathway members (GLI1, Shh and NF-κB) in 51 ductal breast carcinoma specimens by immunohistochemical analysis, and we correlated their expression with clinico-pathological variables. The number of carcinoma specimens and the percentage of tumor cells displaying nuclear GLI1 expression were significantly associated with the tumor grade. Additionally, the percentage of cytoplasmic Shh staining correlated with the tumor grade. These results are consistent with those of other studies that evaluated the clinico-pathological significance of Hh signaling in breast tumors. Cui *et al.* suggested that Shh up-regulation may be an early event in breast carcinogenesis because Shh expression was elevated in early-stage breast carcinoma [19]. In addition, increases in Hh ligand expression and Hh pathway activation have been reported over the progression of malignant breast epithelial lesions from non-neoplastic masses to ductal carcinoma *in situ* (DCIS) to invasive ductal carcinoma (IDC) [17, 35]. Furthermore, we evaluated the cytoplasmic expression of NF-κB and found a positive correlation between the number of NF-κB-positive carcinoma specimens and smaller tumor size. Nakashima *et al*. reported that NF-κB can directly induce Shh expression *in vitro* and *in vivo* and can promote pancreatic cancer cell proliferation and apoptosis resistance *via* the Shh pathway [21, 36]. Interestingly, the percentage of cells displaying nuclear GLI1 staining positively correlated with ErbB2 expression in carcinoma samples (*p* = 0.027), and this result suggests the involvement of Hh pathway activation in the development of ErbB2-positive breast cancer. Thus, the Hh signaling pathway may be a potential therapeutic target for these types of breast cancer. In fact, Hh signaling can be blocked at many levels using a variety of small molecules that target different members of this pathway. More than 50 compounds have been identified as inhibitors of Hh signaling in cancer [22]. In particular, GDC-0449 (Vismodegib/Erivedge^TM^), an SMO antagonist, entered clinical trials and was approved in January 2012 by the FDA for the treatment of adults with locally advanced or metastatic BCC who cannot be treated with surgery or radiation [37]. Another promising therapeutic agent is GANT-61, which directly inhibits the transcription factor GLI, and the efficacy of GANT-61 in blocking the Hh pathway is under investigation in many preclinical studies [38-40].

In light of these findings, we evaluated the *in vitro* anti-cancer activities of GDC-0449 and GANT-61 in human breast cancer cell lines and murine TUBO cells. GANT-61 was more effective than GDC-0449 in reducing the survival of breast cancer cells. In particular, GANT-61 at doses ranging from 5 to 20 μM significantly reduced the survival of 7/8 tested cell lines after 48 hours and in all cell lines after 72 hours of treatment relative to the vehicle control. GDC-0449 significantly decreased cell survival in all cell lines only at the highest dose (12 μM) after 72 hours, 96 hours or 6 days of treatment. In addition, GANT-61 treatment induced apoptosis and altered the cell cycle distribution more effectively than GDC-0449. Specifically, GANT-61 increased the percentage of cells in sub-G1 phase, in all breast cancer cell lines investigated after 48 hours, whereas GDC-0449 exhibited this effect only in 4 cell lines after 6 days of treatment.

The activation of apoptosis by GDC-0449 and GANT-61 was confirmed by PARP-1 cleavage or down-regulation and/or an increase in the ratio of the pro-apoptotic protein Bax to the anti-apoptotic protein Bcl-2.

In addition, GANT-61 inhibited Hh pathway activity more potently than GDC-0449, as demonstrated by the decreased mRNA levels of GLI1 and Ptch after treatment with these compounds. Hh pathway activation leads to the transcription of Hh target gene products, including ubiquitous genes such as GLI1 and Ptch1 and cell-specific genes [3]. Furthermore, based on immunofluorescence analysis, GANT-61 inhibited the translocation of GLI1 to the nucleus and thereby blocked the activation of GLI target genes. After GANT-61 treatment, nuclear GLI1 expression was reduced in MCF-7 and SK-BR-3 cells.

These findings suggest the importance of targeting the Hh pathway using antagonists that act downstream of SMO [41]. This is a more efficient strategy for interrupting Hh signaling considering the presence of SMO mutations and of the “non-canonical” mechanism of GLI protein activation in cancer. Recent studies have shown that mutant forms of SMO are resistant to SMO antagonists such as GDC-0449 [42-44]. Other studies reported that GANT-61 inhibits GLI1/GLI2 in colon cancer cells that are resistant to the SMO inhibitors cyclopamine and GDC-0449; this observation suggests SMO-independent Hh signaling pathway activation in colon cancer [38].

Thus, the “non-canonical” activation of the Hh signaling pathway in cancer leads to the transcription of GLI target genes in a SMO-independent manner *via* alternative signaling pathways. All “non-canonical” activation mechanisms described herein act together with the Hh pathway downstream of SMO. For example, the Ras and TGF-β pathways induce GLI gene activity in a ligand-independent manner [45]. Furthermore, accumulating evidence indicate that a complex interplay can occur between the ErbB receptor, Hh and NF-κB pathways. Therefore, we also investigated the effects of GDC-0449 and GANT-61 on the ErbB receptor and NF-κB signaling pathways in breast cancer cell lines. The responses of the cells to GDC-0449 or GANT-61 treatment varied according to the cell line. Indeed, breast cancer cell lines display different receptor (ER, PR, ErbB2) expression profiles and different levels of normal and mutated signaling proteins (p53, Ras) [26-31]. Intra-tumor and inter-tumor heterogeneity are features of neoplasia. Established human cell lines are derived from human breast cancer tissues or pleural effusions. Thus, breast cancer cell lines display the same genomic alterations as cancer tissues. The responses of such cells lines therefore might predict the resistance of cancer tissues to treatment. Drug resistance might involve a single or multiple signaling pathway alterations [46]. However, no correlation between the drug response and the alteration of any specific signaling pathway was detected. For example, GANT-61 induced PARP-1 cleavage in both MCF-7 and MDA-MB-468 cells, which express wild type and mutated p53, respectively. Activation of the extrinsic or intrinsic apoptotic signaling pathways could be responsible for the observed responses.

GANT-61 altered the protein expression of EGFR and ErbB2. In contrast, GDC-0449 had no effect on the ErbB2 protein level and induced a slight decrease in the EGFR expression level only in T47-D cells. Interestingly, overexpressing *neu* TUBO cells increased EGFR expression, and the *neu* expression level did not change after GANT-61 treatment. A similar result was observed in MDA-MB-468, MCF-7 and BT-474 cells, in which GANT-61 treatment increased the EGFR protein level but had no effect on ErbB2 expression. In these cells, we also observed the induction of apoptosis. Rush *et al*. recently reported that EGFR might trigger apoptosis to prevent prolonged EGFR signaling, which might result from EGFR overexpression or defective EGFR signaling attenuation [47].

In addition, GANT-61 significantly inhibited p21-Ras expression; further studies are needed to elucidate the mechanism by which GANT-61 exerts this effect. MAPKs are the intracellular effectors of ErbB receptors (EGFR, ErbB2, ErbB3 and ErbB4), and hyperactivation of the ERK1/2 MAPK signaling pathway frequently occurs in human cancer and is associated with increased cell survival and proliferation [48]. Here, we demonstrate that MAPK activity was down-regulated by GANT-61 in breast cancer cells. In particular, GANT-61 treatment inhibited ERK1/2 activation in ER- and PR-negative breast cancer cells (MDA-MB-231, MDA-MB-468 and MDA-MB-453 cells) and completely abolished ERK1/2 activation in TUBO cells, leading to the overexpression of ErbB2/*neu*. In contrast, GDC-0449 reduced the p-ERK1/2 levels only in MCF-7 cells. GANT-61 also inhibited the phosphorylation and activation of the pro-survival kinase AKT, which induces tumor growth. Indeed, AKT promotes cell survival and apoptosis resistance by sequestering various protein targets, including the FOXO family of forkhead transcription factors and the pro-apoptotic protein Bad, and by activating the pro-survival transcription factor NF-κB [49]. However, GANT-61 inhibited NF-κB activity by blocking the nuclear translocation of NF-κB in MCF-7 and SK-BR-3 cells.

Overall, our results suggest that both GDC-0449 and GANT-61 control Hh/ErbB/NF-κB signaling pathways *in vitro*, although the GLI1 inhibitor was more effective than the SMO inhibitor. Several studies and phase I clinical trials have shown that GDC-0449 has a good human safety profile, low plasma clearance, good oral bioavailability (13-53%), metabolic stability in hepatocytes, good absorption and wide distribution to tissues [50-52]. GDC-0449 was administered at a dose of 150 mg daily, and its maximum plasma concentrations were approximately 3.58 μM and 23 μM for single and repeated doses, respectively [50-53]. Accordingly, the GDC-0449 doses that we employed in our *in vitro* experiments (3-25 μM) are in agreement with its plasma concentrations in treated patients.

In addition, GDC-0449 has been effective in the treatment of solid organ tumors such as BCC and medulloblastoma [53, 54]. With respect to its efficacy in mouse models, GDC-0449 (12.5 mg/kg) has been shown to induce complete tumor regression in an allograft mouse model of medulloblastoma that is entirely dependent on the Hh pathway for growth [23]. GDC-0449 significantly reduced liver fibrosis and the tumor burden in aged Mdr2-deficient mice that already exhibited advanced liver fibrosis and hepatocellular carcinoma [55] and blocked the growth of tamoxifen-resistant breast cancer cell xenografts in mice [56].

Concerning GANT-61, a recent study reported the *in vivo* antitumor potential of this Hh inhibitor in neuroblastoma. Mice subcutaneously injected into the flank with neuroblastoma cells were treated orally through gastric feeding with GANT-61 (50 mg/kg), and a reduction of 63% in tumor volume was observed in comparison with the control treatment [57].

Given these observations, we evaluated the *in vivo* therapeutic potential of these two Hh inhibitors in BALB/*c* mice that were subcutaneously inoculated into the right flank with TUBO cells and simultaneously treated *per os* with 2 mg of GDC-0449, GANT-61 or vehicle alone per mouse. Overall, our results indicated that the Hh inhibitors specifically interfered with TUBO tumor cell growth to varying extents. Indeed, considering the survival of BALB/*c* mice, the risk of TUBO tumor cell growth in the corn oil-treated group was 0.016 and 49.40 relative to the GDC-0449 and GANT-61-treated groups, respectively. In addition, the risk of TUBO tumor cell growth in the GDC-0449-treated group was 17.81 relative to the GANT-61-treated group. Mice treated with GDC-0449 or GANT-61 showed a significant decrease in the mean tumor volume compared with control mice (48% decrease for GDC-0449, 75% decrease for GANT-61) after 3 weeks of treatment. GDC-0449 increased the survival of BALB/*c* mice by only one week compared with the control mice (*p* = 0.0009). In contrast, TUBO cell growth was counteracted in mice treated with GANT-61; in 4/5 mice treated with GANT-61, we observed complete tumor regression after 6 weeks of treatment.

Our results provide evidence that GDC-0449 and GANT-61 suppress the growth of human and mouse breast cancer cells *in vitro* and *in vivo,* although to different extents. However, the GLI1 inhibitor blocked the Hh pathway more effectively than the SMO inhibitor, likely because cancer cells exhibit “non-canonical” activation of the Hh pathway, which is caused by alternative signaling pathways (ErbB receptors, NF-κB, Wnt, and Notch) [58]. To date, SMO inhibitors have mainly been employed to target Hh signaling. However, resistance to the presently employed SMO inhibitors has been found in BCC patients. Thus, drugs directly inhibiting GLI transcription factors will also be helpful in the treatment of tumors harboring mutations in SMO or in factors downstream of SMO (e.g., SuFu) [41].

Overall, GANT61 inhibits cell growth, DNA damage repair, epithelial-mesenchymal transition, cancer stem cell survival and induces apoptosis, autophagy and inflammatory responses [41]. Accordingly, it is important to develop new breast cancer therapies based on the combination of Hh pathway inhibitors and ErbB2, EGFR, Wnt and Notch signaling inhibitors.

## MATERIALS AND METHODS

### Reagents

DMSO, GANT-61, Sulforhodamine B (SRB) and Hoechst 33342 were purchased from Sigma Aldrich (Milano, Italy). GDC-0449 (CUR-0199691) was provided by Genentech (San Francisco, CA). Antibodies against AKT, and phospho-AKT were obtained from Cell Signaling Technology (MA, USA). Antibodies against Bax and Bcl-2 were from BD Pharmigen (BD Biosciences, San Josè, CA, USA). Antibodies against GLI1 (H-300), Shh (H-160), ERK1/2 (C-14), phospho-ERK (E-4), NF-κB (p65), PARP-1, p53 (DO-1) and H-Ras (259) were obtained from Santa Cruz Biotechnology (CA, USA). Anti-ErbB2 and anti-EGFR antisera were provided by Dr. M.H. Kraus (University of Alabama, Birmingham, USA). Goat anti-rabbit IgG Alexa fluor-594-conjugated and goat anti-mouse IgG Alexa fluor-488-conjugated secondary antibody came from Invitrogen (Milano, Italy). A rabbit polyclonal antibody against actin and goat anti-mouse or anti-rabbit IgG peroxidase-conjugated secondary antibodies were from Sigma-Aldrich.

### Tissue micro-array (TMA) and immunohistochemistry

The expression of different proteins in human tissues was determined by immunoperoxidase staining after incubation with specific antibodies.

We analyzed 51 invasive ductal breast carcinoma specimens including the following: G1 = n.10, G2 = n.31, and G3 = n.10. Patient tissue was obtained according to the ethical guidelines of the Policlinico of Tor Vergata, Rome.

For TMA construction, we used tissue fragments left over from sampling procedures that were employed for diagnostic purposes. Areas of interest from breast carcinoma specimens were identified on corresponding H&E-stained sections and marked on the donor paraffin block. A 3-mm-thick core of the donor block was placed in the recipient master block of a Galileo TMA CK2500 (Brugherio, Milano, Italy). Three cores from different areas of the same tissue block were arrayed for each case (the total amount of neoplastic cells was not less than 1500) [59]. For immunohistochemistry, 4-μm paraffin sections from TMA were deparaffinized, rehydrated and quenched in a 0.2% hydrogen peroxide solution diluted in methanol. Nonspecific sites were blocked for 5 min in a buffer containing 100 mM Tris, BSA 2% horse serum, and 0.02% sodium azide. After pretreatment for 30 min at 100°C in EDTA citric buffer, the sections were immunolabeled for 1 hour at room temperature with the primary antibodies anti-ER (prediluted; rabbit monoclonal antibody SP1; Ventana, Tucson, AZ USA), anti-PR (prediluted; rabbit monoclonal antibody 1E2; Ventana, Tucson, AZ USA), anti-Her2/neu (prediluted; rabbit monoclonal antibody 4B5; Ventana, Tucson, AZ USA), anti-EGFR (prediluted; rabbit monoclonal antibody 5B7; Ventana, Tucson, AZ USA), anti-NF-κB (1:100 diluted; mouse polyclonal antibody; Santa Cruz Biotechnology, CA, USA), anti-GLI1 (1:100 diluted; rabbit polyclonal antibody; Santa Cruz Biotechnology, CA, USA) and anti-Shh (1:200 diluted; rabbit polyclonal antibody; Santa Cruz Biotechnology, CA, USA). This step was followed by a 15 min incubation with biotinylated anti-rabbit/mouse IgG (prediluted; Dako Denmark, Glostrup Denmark) and by a 15-min incubation with avidin-peroxidase complex (prediluted; Dako Denmark, Glostrup Denmark). The reactions were revealed with a Universal DAB Detection Kit (Dako Denmark, Glostrup Denmark) [60].

The semiquantitative expression of GLI1, Shh, and NF-κB in the carcinoma specimens was estimated at x200 magnification in at least 10 fields by two investigators in a blind fashion. A previously described score system with minor modifications was used. The staining intensity was semi-quantitatively classified as negative (−), weakly positive (+), and overexpressed (2+ and 3+). The percentage of cells with positive staining was assessed independently. A tumor was considered negative for ER and PR expression if < 5% of tumor cell nuclei were stained. The HER2 expression level was scored on the basis of membrane staining intensity as follows: 0 (negative) and 1 + (discontinuous and weak) staining were considered negative, 2 + (continuous and weak to moderate) staining was considered borderline, and 3 + (continuous and strong) was considered positive. Borderline cases have been solved by FISH analysis. Accordingly, the presence of less than two copies of the HER2 gene was scored as negative, and that of two or more copies was scored as positive. Nuclear grading was assessed by evaluating the nuclear size, monomorphism or pleomorphism, the dispersion of chromatin, and the number of nucleoli and mitosis, and they were scored as low (G1), intermediate (G2) and high (G3) [61].

### Cell lines and treatments

BALB-*neu*T mammary cancer cells (H-2^d^) (TUBO) that overexpress activated rat ErbB2/*neu* were kindly provided by Prof. G. Forni (University of Torino, Italy) [62]. Human (MDA-MB-231, MDA-MB-453, MDA-MB-468, T47-D, MCF-7, BT-474, and SK-BR-3) and mouse breast cancer cell lines (TUBO) and human MCF-10A mammary epithelial cells were maintained in DMEM (Dulbecco's modified Eagle's medium) containing 10% fetal bovine serum, 100 U/ml penicillin and 100 μg/ml streptomycin (complete medium). The cells were grown at 37°C in a humidified incubator with an atmosphere of 5% CO_2_.

GDC-0449 and GANT-61 were dissolved in DMSO. For the treatments, the cells were incubated for the indicated times in the presence of GDC-0449 (dose range: 3-25 μM), GANT-61 (dose range: 1-20 μM) or a vehicle control (DMSO ≤ 0.1%).

### Sulforhodamine B (SRB) assay

Cells were seeded at 5×10^3^/well in 96-well plates and incubated at 37°C to allow cell attachment. After 24 hours, the medium was changed and the cells were treated with GDC-0449 or DMSO and incubated for 48 hours, 72 hours, 96 hours or 6 days at concentrations of 3-6-12 μM, or with GANT-61 or DMSO and then incubated for 48 and 72 hours at concentrations of 1-2.5-5-10-20 μM. The cells were then fixed with cold trichloroacetic acid (final concentration 10%) for 1 h at 4°C. After 4 washes with distilled water, the plates were air-dried and stained for 30 min with 0.4% (wt/vol) SRB in 1% acetic acid. After 4 washes with 1% acetic acid to remove the unbound dye, the plates were air-dried, and cell-bound SRB was dissolved with 200 μl/well of 10 mM unbuffered Tris base solution. The optical density (O.D.) of the samples was determined at 540 nm with a spectrophotometric plate reader. The percentage survival of the cultures treated with GDC-0449 or GANT-61 was calculated by normalizing their O.D. values to those of control cultures treated with DMSO [63]. The experiments were performed in triplicate and repeated three times.

### FACS analysis

Asynchronized, log-phase growing cells (60% confluent, approximately 2.5 × 10^5^/well in 6-well plates) were treated with GDC-0449, GANT-61 or DMSO in complete culture medium. After 96 hours or 6 days for GDC-0449 (3-6-12-25 μM) or 48 hours for GANT-61 (5-10-20 μM), adherent as well as suspended cells were harvested, centrifuged at 1500 rpm for 10 min and washed twice with cold phosphate-buffered saline (PBS). The cell pellets were resuspended in 70% ethanol and incubated for 1 hour at −20°C. The cells were then washed twice with cold PBS, centrifuged at 1500 rpm for 10 min, incubated for 1 hour in the dark with propidium iodide (25 μg/ml final concentration in 0.1% citrate and 0.1% Triton X-100) and analyzed by flow cytometry using a FACSCalibur cytometer with CellQuest software [49].

### RNA isolation and real-time q-PCR

Cells were seeded at 2 × 10^5^ cells/well in 6-well plates and incubated at 37°C to allow cell attachment. After 24 hours, the cells were treated with GDC-0449 (50 μM for 48 h; 12-25 μM for 24 h), GANT-61 (20 μM for 48 h) or DMSO. After incubation, the cells were harvested, washed twice with PBS and centrifuged at 1200 rpm for 10 min.

The total RNA from the cells was extracted using TRI Reagent Solution (Ambion^TM^, Life Technologies, Italy), according to the manufacturer's instructions, and cDNA synthesis was performed with a SuperScript II First-Strand Synthesis kit (Invitrogen, Life Technologies, Italy). A quantitative real-time PCR analysis of *hGLI1, hGLI2, hIkbα, hPtch1, mGLI1, mGLI2, mPtch1, mIkbα, hHPRT, hB2M, hTBP, mHPRT, mB2M,* and *mTBP* messenger RNA (mRNA) was performed on cDNA using TaqMan gene expression assays according to the manufacturer's instructions (Applied Biosystems, Life Technologies, Italy) and an ABI Prism 7900HT (Applied Biosystems, Life Technologies, Italy) as previously described [64]. Each amplification reaction was performed in triplicate, and the average of the three threshold cycles was used to calculate the amount of transcripts in the sample (SDS software; ABI). mRNA quantification was expressed, in arbitrary units, as the ratio of the sample quantity to the calibrator or to the mean values of the control samples. All values were normalized to three endogenous controls, namely *HPRT, B2M*, and *TBP.*

### Preparation of cell lysates and Western blotting

Approximately 1 × 10^6^ cells were seeded in 100-mm tissue culture dishes 24 hours prior to the addition of 12 μM GDC-0449, 10 μM GANT-61 or vehicle control. After 48 hours (for GANT-61) or 6 days (for GDC-0449) of incubation, the cells were harvested, washed twice with cold PBS and lysed in RIPA lysis buffer (Triton X-100 1%, SDS 0.1%, NaCl 200 mM, Tris HCl 50 mM pH 7.5, PMSF 1 mM, and NaOV 1 mM). After 30 min at 4°C, the mixtures were centrifuged at 12000 g for 15 min and the supernatants were analyzed by Western blotting. For immunoblotting analysis, 50 μg of cell lysates were resolved in 10% SDS-PAGE and then transferred to nitrocellulose membranes. After blocking, the membranes were incubated with specific primary antibodies at 1-2 μg/ml concentrations overnight at 4°C. After being washed, the filters were incubated with goat anti-mouse or anti-rabbit IgG, peroxidase-conjugated antibodies and developed by chemiluminescence as previously described [65]. A densitometric analysis of autoradiographic bands was performed with Image J software (National Institutes of Health, USA) after blot scanning.

### Immunofluorescence

Cells were seeded at 4 × 10^4^ cells/well in 8-well chamber slides and, after 24 hours, they were treated with 10 μM GANT, or with vehicle control. After 24 hours, the cells were fixed in 4% formaldehyde for 10 min, washed and fixed in methanol for 5 min at −20°C, then washed and incubated with GLI1 polyclonal antibody overnight at 4°C or with NF-κB monoclonal antibody for 1 hour at room temperature. After additional washings, the cells were labeled with a goat anti-rabbit IgG Alexa fluor-594-conjugated antibody for 30 min [66]. After a third washing, the cells were incubated with 0.1 μg/ml Hoechst 33342 and mounted under a cover slip with glycerol. The cells were observed with an Olympus BX51 microscope.

### *In vivo* treatment of BALB/*c* mice with GDC-0449 or GANT-61

Groups of 6-to-8-week-old BALB/*c* female mice (5 mice per group) were subcutaneously inoculated in the right flank with 0.2 ml of suspension containing 1×10^6^ TUBO cells in phosphate-buffered saline (PBS) and treated *per os* with GDC-0449 (2 mg dissolved in 100 μl of corn oil-ethanol in a 4:1 ratio, 3 times per week), GANT-61 (2 mg dissolved in 100 μl of corn oil-ethanol in a 4:1 ratio, 1 time per week) or corn oil-ethanol (100 μl in a 4:1 ratio, 3 times per week). The treatments were started simultaneously with the inoculation of TUBO cells.

Investigation has been conducted in accordance with the ethical standards and according to the Declaration of Helsinki. A veterinary surgeon was present during the experiments. The animal care both before and after the experiments was performed only by trained personnel. Mice were bred under pathogen-free conditions in the animal facilities of Tor Vergata University and handled in compliance with European Union and institutional standards for animal research.

### Analysis of antitumor activity *in vivo*

Tumor growth was monitored weekly until tumor-bearing mice were sacrificed at the first signs of distress or when their tumors exceeded 20 mm in diameter [67]. The tumors were measured with a caliper in two dimensions and the volumes were calculated by using the following formula: width^2^ x length/2 [68].

### Statistical analysis

Differences in GLI1, Shh and NF-κB expression and their association with clinico-pathological parameters were evaluated using Fisher's exact test and Student's *t*-test. Values with p≤ 0.05 were considered significant. The data distribution of cell survival and the FACS analyses were preliminarily verified by Kolmogorov-Smirnov test, and data sets were analyzed by one-way analysis of variance (ANOVA) followed by Newman-Keuls test. Differences in GLI1 or Ptch mRNA expression levels and the intensity of immunoreactive bands were evaluated by a two-tailed Student's *t*-test. Values with p≤ 0.05 were considered significant. Survival curves and tumor multiplicity were estimated by Kaplan-Meier method and compared by log-rank test (Mantel-Cox). Differences in tumor volumes were regarded as significant when the p value was ≤ 0.05 [66].

### Abbreviations

HH/GLI, Hedgehog (Hh)/Glioma-associated oncogene (GLI); Shh, Sonic Hedgehog; Ptch, Patched-1; SMO, Smoothened; EGFR, epidermal growth factor receptor; ER, estrogen receptor; PR, progesterone receptor; DMSO, Dimethyl sulfoxide; PARP-1, Poly (ADP-ribose) polymerase-1; ERK, extracellular signal-related kinase; p-ERK, phospho-ERK; p-AKT, phospho-AKT; MAPK, mitogen-activated protein kinase.

## SUPPLEMENTARY MATERIAL FIGURE AND TABLES



## References

[1] Jiang J, Hui CC (2008). Hedgehog signaling in development and cancer. Dev Cell.

[2] Varjosalo M, Taipale J (2008). Hedgehog: functions and mechanisms. Genes Dev.

[3] Gupta S, Takebe N, Lorusso P (2010). Targeting the Hedgehog pathway in cancer. Ther Adv Med Oncol.

[4] Benvenuto M, Fantini M, Masuelli L, De Smaele E, Zazzeroni F, Tresoldi I, Calabrese G, Galvano F, Modesti A, Bei R (2013). Inhibition of ErbB receptors, Hedgehog and NF-kappaB signaling by polyphenols in cancer. Front Biosci (Landmark Ed).

[5] Scales SJ, de Sauvage FJ (2009). Mechanisms of Hedgehog pathway activation in cancer and implications for therapy. Trends Pharmacol Sci.

[6] Varjosalo M, Taipale J (2007). Hedgehog signaling. J Cell Sci.

[7] Ferretti E, De Smaele E, Di Marcotullio L, Screpanti I, Gulino A (2005). Hedgehog checkpoints in medulloblastoma: the chromosome 17p deletion paradigm. Trends Mol Med.

[8] De Smaele E, Di Marcotullio L, Moretti M, Pelloni M, Occhione MA, Infante P, Cucchi D, Greco A, Pietrosanti L, Todorovic J, Coni S, Canettieri G, Ferretti E, Bei R, Maroder M, Screpanti I, Gulino A (2011). Identification and characterization of KCASH2 and KCASH3, 2 novel Cullin3 adaptors suppressing histone deacetylase and Hedgehog activity in medulloblastoma. Neoplasia.

[9] Canettieri G, Di Marcotullio L, Greco A, Coni S, Antonucci L, Infante P, Pietrosanti L, De Smaele E, Ferretti E, Miele E, Pelloni M, De Simone G, Pedone EM, Gallinari P, Giorgi A, Steinkühler C, Vitagliano L, Pedone C, Schinin ME, Screpanti I, Gulino A (2010). Histone deacetylase and Cullin3-REN(KCTD11) ubiquitin ligase interplay regulates Hedgehog signalling through Gli acetylation. Nat Cell Biol.

[10] Coni S, Antonucci L, D'Amico D, Di Magno L, Infante P, De Smaele E, Giannini G, Di Marcotullio L, Screpanti I, Gulino A, Canettieri G (2013). Gli2 acetylation at lysine 757 regulates hedgehog-dependent transcriptional output by preventing its promoter occupancy. PLoS One.

[11] Kubo M, Nakamura M, Tasaki A, Yamanaka N, Nakashima H, Nomura M, Kuroki S, Katano M (2004). Hedgehog signaling pathway is a new therapeutic target for patients with breast cancer. Cancer Res.

[12] Xie J, Johnson RL, Zhang X, Bare JW, Waldman FM, Cogen PH, Menon AG, Warren RS, Chen LC, Scott MP, Epstein EH (1997). Mutations of the PATCHED gene in several types of sporadic extracutaneous tumors. Cancer Res.

[13] Vorechovsky I, Benediktsson KP, Toftgard R (1999). The patched/hedgehog/smoothened signalling pathway in human breast cancer: no evidence for H133Y SHH, PTCH and SMO mutations. Eur J Cancer.

[14] Wicking C, Evans T, Henk B, Hayward N, Simms LA, Chenevix-Trench G, Pietsch T, Wainwright B (1998). No evidence for the H133Y mutation in SONIC HEDGEHOG in a collection of common tumour types. Oncogene.

[15] Jiao X, Wood LD, Lindman M, Jones S, Buckhaults P, Polyak K, Sukumar S, Carter H, Kim D, Karchin R, Sjöblom T (2012). Somatic mutations in the Notch, NF-kB, PIK3CA, and Hedgehog pathways in human breast cancers. Genes Chromosomes Cancer.

[16] Mukherjee S, Frolova N, Sadlonova A, Novak Z, Steg A, Page GP, Welch DR, Lobo-Ruppert SM, Ruppert JM, Johnson MR, Frost AR (2006). Hedgehog signaling and response to cyclopamine differs in epithelial and stromal cells in benign breast and breast cancer. Cancer Biol Ther.

[17] O'Toole SA, Machalek DA, Shearer RF, Millar EK, Nair R, Schofield P, McLeod D, Cooper CL, McNeil CM, McFarland A, Nguyen A, Ormandy CJ, Qiu MR (2011). Hedgehog overexpression is associated with stromal interactions and predicts for poor outcome in breast cancer. Cancer Res.

[18] Wolf I, Bose S, Desmond JC, Lin BT, Williamson EA, Karlan BY, Koeffler HP (2007). Unmasking of epigenetically silenced genes reveals DNA promoter methylation and reduced expression of PTCH in breast cancer. Breast Cancer Res Treatment.

[19] Cui W, Wang LH, Wen YY, Song M, Li BL, Chen XL, Xu M, An SX, Zhao J, Lu YY, Mi XY, Wang EH (2010). Expression and regulation mechanisms of Sonic Hedgehog in breast cancer. Cancer Sci.

[20] Mangelberger D, Kern D, Loipetzberger A, Eberl M, Aberger F (2012). Cooperative Hedgehog-EGFR signaling. Front Biosci.

[21] Nakashima H, Nakamura M, Yamaguchi H, Yamanaka N, Akiyoshi T, Koga K, Yamaguchi K, Tsuneyoshi M, Tanaka M, Katano M (2006). Nuclear factor-kappaB contributes to hedgehog signaling pathway activation through sonic hedgehog induction in pancreatic cancer. Cancer Res.

[22] Hadden MK (2013). Hedgehog pathway inhibitors: a patent review (2009—present). Expert Opin Ther Pat.

[23] Robarge KD, Brunton SA, Castanedo GM, Cui Y, Dina MS, Goldsmith R, Gould SE, Guichert O, Gunzner JL, Halladay J, Jia W, Khojasteh C, Koehler MF (2009). GDC-0449-a potent inhibitor of the hedgehog pathway. Bioorg Med Chem Lett.

[24] De Smaele E, Ferretti E, Gulino A (2010). Vismodegib, a small-molecule inhibitor of the hedgehog pathway for the treatment of advanced cancers. Curr Opin Investig Drugs.

[25] Lauth M, Bergstrom A, Shimokawa T, Toftgard R (2007). Inhibition of GLI-mediated transcription and tumor cell growth by small-molecule antagonists. Proc Natl Acad Sci U S A.

[26] Neve RM, Chin K, Fridlyand J, Yeh J, Baehner FL, Fevr T, Clark L, Bayani N, Coppe JP, Tong F, Speed T, Spellman PT, De Vries S (2006). A collection of breast cancer cell lines for the study of functionally distinct cancer subtypes. Cancer Cell.

[27] Charafe-Jauffret E, Ginestier C, Monville F, Finetti P, Adélaïde J, Cervera N, Fekairi S, Xerri L, Jacquemier J, Birnbaum D, Bertucci F (2006). Gene expression profiling of breast cell lines identifies potential new basal markers. Oncogene.

[28] Eckert LB, Repasky GA, Ülkü AS, McFall A, Zhou H, Sartor CI, Der CJ (2004). Involvement of Ras activation in human breast cancer cell signaling, invasion, and anoikis. Cancer Research.

[29] Lacroix M, Toillon RA, Leclercq G (2006). p53 and breast cancer, an update. Endocr Relat Cancer.

[30] Chavez KJ, Garimella SV, Lipkowitz S (2010). Triple negative breast cancer cell lines: one tool in the search for better treatment of triple negative breast cancer. Breast Dis.

[31] Vranic S, Gatalica Z, Wang ZY (2011). Update on the molecular profile of the MDA-MB-453 cell line as a model for apocrine breast carcinoma studies. Oncology Letters.

[32] Olayioye MA, Neve RM, Lane HA, Hynes NE (2000). The ErbB signaling network: receptor heterodimerization in development and cancer. EMBO J.

[33] McKay MM, Morrison DK (2007). Integrating signals from RTKs to ERK/MAPK. Oncogene.

[34] Visbal AP, Lewis MT (2010). Hedgehog signaling in the normal and neoplastic mammary gland. Curr Drug Targets.

[35] Xuan Y, Lin Z (2009). Expression of Indian Hedgehog signaling molecules in breast cancer. J Cancer Res Clin Oncol.

[36] Kasperczyk H, Baumann B, Debatin KM, Fulda S (2009). Characterization of sonic hedgehog as a novel NF-kappaB target gene that promotes NF-kappaB-mediated apoptosis resistance and tumor growth *in vivo*. FASEB J.

[37] FDA approval for vismodegib http://www.cancer.gov/cancertopics/druginfo/fda-vismodegib.

[38] Agyeman A, Mazumdar T, Houghton JA (2012). Regulation of DNA damage following termination of Hedgehog (HH) survival signaling at the level of the GLI genes in human colon cancer. Oncotarget.

[39] You M, Varona-Santos J, Singh S, Robbins DJ, Savaraj N, Nguyen DM (2014). Targeting of the Hedgehog signal transduction pathway suppresses survival of malignant pleural mesothelioma cells *in vitro*. J Thorac Cardiovasc Surg.

[40] Srivastava RK, Kaylani SZ, Edrees N, Li C, Talwelkar SS, Xu J, Palle K, Pressey JG, Athar M (2014). GLI inhibitor GANT-61 diminishes embryonal and alveolar rhabdomyosarcoma growth by inhibiting Shh/AKT-mTOR axis. Oncotarget.

[41] Gonnissen A, Isebaert S, Haustermans K (2015). Targeting the Hedgehog signaling pathway in cancer: beyond Smoothened. Oncotarget.

[42] Yauch RL, Dijkgraaf GJ, Alicke B, Januario T, Ahn CP, Holcomb T, Pujara K, Stinson J, Callahan CA, Tang T, Bazan JF, Kan Z, Seshagiri S, Hann CL, Gould SE, Low JA, Rudin CM, de Sauvage FJ (2009). Smoothened mutation confers resistance to a Hedgehog pathway inhibitor in medulloblastoma. Science.

[43] Dijkgraaf GJ, Alicke B, Weinmann L, Januario T, West K, Modrusan Z, Burdick D, Goldsmith R, Robarge K, Sutherlin D, Scales SJ, Gould SE, Yauch RL, de Sauvage FJ (2011). Small molecule inhibition of GDC-0449 refractory smoothened mutants and downstream mechanisms of drug resistance. Cancer Res.

[44] Buonamici S, Williams J, Morrissey M, Wang A, Guo R, Vattay A, Hsiao K, Yuan J, Green J, Ospina B, Yu Q, Ostrom L, Fordjour P (2010). Interfering with resistance to smoothened antagonists by inhibition of the PI3K pathway in medulloblastoma. Sci Transl Med.

[45] Lauth M, Toftgård R (2007). Non-canonical activation of GLI transcription factors: implications for targeted anti-cancer therapy. Cell Cycle.

[46] Koren S, Bentires-Alj M (2015). Breast Tumor Heterogeneity: Source of fitness, hurdle for therapy. Mol Cell.

[47] Rush JS, Quinalty LM, Engelman L, Sherry DM, Ceresa BP (2012). Endosomal accumulation of the activated epidermal growth factor receptor (EGFR) induces apoptosis. J Biol Chem.

[48] Masuelli L, Benvenuto M, Fantini M, Marzocchella L, Sacchetti P, Di Stefano E, Tresoldi I, Izzi V, Bernardini R, Palumbo C, Mattei M, Lista F, Galvano F, Modesti A, Bei R (2013). Curcumin induces apoptosis in breast cancer cell lines and delays the growth of mammary tumors in neu transgenic mice. J Biol Regul Homeost Agents.

[49] Masuelli L, Marzocchella L, Focaccetti C, Tresoldi I, Palumbo C, Izzi V, Benvenuto M, Fantini M, Lista F, Tarantino U, Modesti A, Galvano F, Bei R (2012). Resveratrol and diallyl disulfide enhance curcumin-induced sarcoma cell apoptosis. Front Biosci.

[50] Wong H, Chen JZ, Chou B, Halladay JS, Kenny JR, La H, Marsters JC, Plise E, Rudewicz PJ, Robarge K, Shin Y, Wong S, Zhang C, Khojasteh SC (2009). Preclinical assessment of the absorption, distribution, metabolism and excretion of GDC-0449 (2-chloro-N-(4-chloro-3-(pyridin-2-yl)phenyl)-4-(methylsulfonyl)benzamide), an orally bioavailable systemic Hedgehog signalling pathway inhibitor. Xenobiotica.

[51] Yue Q, Chen YH, Mulder T, Deese A, Takahashi R, Rudewicz PJ, Reynolds M, Solon E, Hop CE, Wong H, Khojasteh SC (2011). Absorption, distribution, metabolism, and excretion of [14C]GDC-0449 (vismodegib), an orally active hedgehog pathway inhibitor, in rats and dogs: a unique metabolic pathway *via* pyridine ring opening. Drug Metab Dispos.

[52] Proctor AE, Thompson LA, O'Bryant CL (2014). Vismodegib: an inhibitor of the Hedgehog signaling pathway in the treatment of basal cell carcinoma. Ann Pharmacother.

[53] LoRusso PM, Rudin CM, Reddy JC, Tibes R, Weiss GJ, Borad MJ, Hann CL, Brahmer JR, Chang I, Darbonne WC, Graham RA, Zerivitz KL, Low JA, Von Hoff DD (2011). Phase I trial of hedgehog pathway inhibitor vismodegib (GDC-0449) in patients with refractory, locally advanced or metastatic solid tumors. Clin Cancer Res.

[54] Rudin CM, Hann CL, Laterra J, Yauch RL, Callahan CA, Fu L, Holcomb T, Stinson J, Gould SE, Coleman B, LoRusso PM, Von Hoff DD, de Sauvage FJ, Low JA (2009). Treatment of medulloblastoma with hedgehog pathway inhibitor GDC-0449. N Engl J Med.

[55] Philips GM, Chan IS, Swiderska M, Schroder VT, Guy C, Karaca GF, Moylan C, Venkatraman T, Feuerlein S, Syn WK, Jung Y, Witek RP, Choi S, Michelotti GA, Rangwala F, Merkle E, Lascola C, Diehl AM (2011). Hedgehog signaling antagonist promotes regression of both liver fibrosis and hepatocellular carcinoma in a murine model of primary liver cancer. PLoS One.

[56] Ramaswamy B, Lu Y, Teng KY, Nuovo G, Li X, Shapiro CL, Majumder S (2012). Hedgehog signaling is a novel therapeutic target in tamoxifen-resistant breast cancer aberrantly activated by PI3K/AKT pathway. Cancer Res.

[57] Wickström M, Dyberg C, Shimokawa T, Milosevic J, Baryawno N, Fuskevåg OM, Larsson R, Kogner P, Zaphiropoulos PG, Johnsen JI (2013). Targeting the hedgehog signal transduction pathway at the level of GLI inhibits neuroblastoma cell growth *in vitro* and *in vivo*. Int J Cancer.

[58] Kasper M, Jaks V, Fiaschi M, Toftgård R (2009). Hedgehog signalling in breast cancer. Carcinogenesis.

[59] Pilla D, Bosisio FM, Marotta R, Faggi S, Forlani P, Falavigna M, Biunno I, Martella E, De Blasio P, Borghesi S, Cattoretti G (2012). Tissue microarray design and construction for scientific, industrial and diagnostic use. J Pathol Inform.

[60] Bei R, Masuelli L, Moriconi E, Visco V, Moretti A, Kraus MH, Muraro R (1999). Immune responses to all ErbB family receptors detectable in serum of cancer patients. Oncogene.

[61] Marzocchella L, Sini V, Buonomo O, Orlandi A, Masuelli L, Bonanno E, Lista F, Turriziani M, Manzari V, Roselli M, Modesti A, Bei R (2011). Spontaneous immunogenicity of ribosomal P0 protein in patients with benign and malignant breast lesions and delay of mammary tumor growth in P0-vaccinated mice. Cancer Sci.

[62] Rovero S, Amici A, Di Carlo E, Bei R, Nanni P, Quaglino E, Porcedda P, Boggio K, Smorlesi A, Lollini PL, Landuzzi L, Colombo MP, Giovarelli M, Musiani P, Forni G (2000). DNA vaccination against rat her-2/Neu p185 more effectively inhibits carcinogenesis than transplantable carcinomas in transgenic BALB/c mice. J Immunol.

[63] Masuelli L, Di Stefano E, Fantini M, Mattera R, Benvenuto M, Marzocchella L, Sacchetti P, Focaccetti C, Bernardini R, Tresoldi I, Izzi V, Mattei M, Frajese GV, Lista F, Modesti A, Bei R (2014). Resveratrol potentiates the *in vitro* and *in vivo* anti-tumoral effects of curcumin in head and neck carcinomas. Oncotarget.

[64] De Smaele E, Fragomeli C, Ferretti E, Pelloni M, Po A, Canettieri G, Coni S, Di Marcotullio L, Greco A, Moretti M, Di Rocco C, Pazzaglia S, Maroder M, Screpanti I, Giannini G, Gulino A (2008). An integrated approach identifies Nhlh1 and Insm1 as sonic Hedgehog-regulated genes in developing cerebellum and medulloblastoma. Neoplasia.

[65] Masuelli L, Budillon A, Marzocchella L, Mrozek MA, Vitolo D, Di Gennaro E, Losito S, Sale P, Longo F, Ionna F, Lista F, Muraro R, Modesti A, Bei R (2012). Caveolin-1 overexpression is associated with simultaneous abnormal expression of the E-cadherin/α-β catenins complex and multiple ErbB receptors and with lymph nodes metastasis in head and neck squamous cell carcinomas. J Cell Physiol.

[66] Masuelli L, Marzocchella L, Focaccetti C, Lista F, Nardi A, Scardino A, Mattei M, Turriziani M, Modesti M, Forni G, Schlom J, Modesti A, Bei R (2010). Local delivery of recombinant vaccinia virus encoding for neu counteracts growth of mammary tumors more efficiently than systemic delivery in neu transgenic mice. Cancer Immunol Immunother.

[67] Masuelli L, Fantini M, Benvenuto M, Sacchetti P, Giganti MG, Tresoldi I, Lido P, Lista F, Cavallo F, Nanni P, Schlom J, Modesti A, Bei R (2014). Intratumoral delivery of recombinant vaccinia virus encoding for ErbB2/Neu inhibits the growth of salivary gland carcinoma cells. J Transl Med.

[68] Bei R, Guptill V, Masuelli L, Kashmiri SV, Muraro R, Frati L, Schlom J, Kantor J (1998). The use of a cationic liposome formulation (DOTAP) mixed with a recombinant tumor-associated antigen to induce immune responses and protective immunity in mice. J Immunother.

