# Supplementary material for: In vitro and in vivo inhibition of breast cancer cell growth by targeting the Hedgehog/GLI pathway with SMO (GDC-0449) or GLI (GANT-61) inhibitors
Source: Oncotarget. 2016 Jan 28;7(8):9250–70. doi: 10.18632/oncotarget.7062 (PMC4891038; doi:10.18632/oncotarget.7062)
Supplement: Supplementary file 1 [file oncotarget-07-9250-s001.pdf]

In vitro and *in vivo* inhibition of breast cancer cell growth by targeting the Hedgehog/GLI pathway with SMO (GDC-0449) or GLI (GANT-61) inhibitors

Supplementary Material

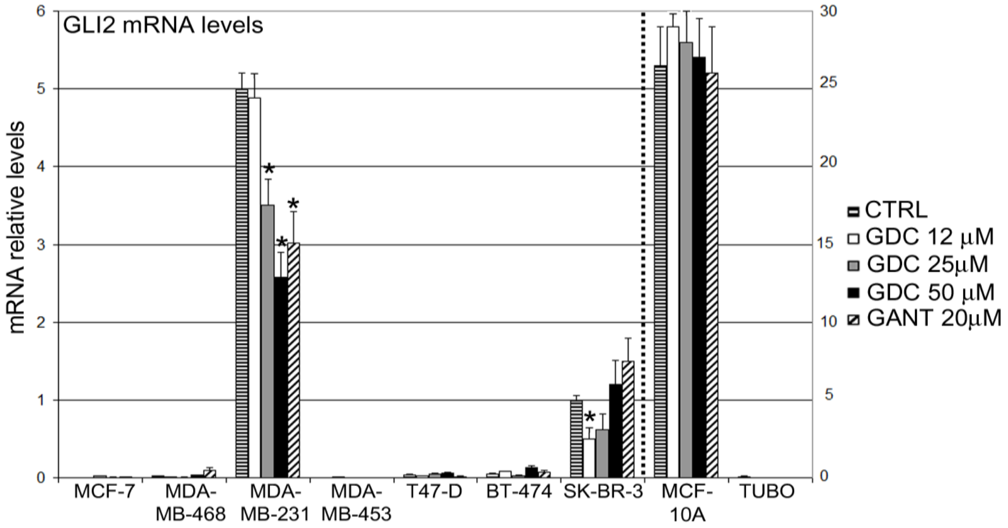

Supplementary Figure 1. The GLI2 mRNA levels in breast cancer cell lines after treatment with GDC-0449 (GDC) or GANT-61 (GANT). Real-time q-PCR was performed to assess the GLI2 mRNA levels in RNA that was isolated from breast cancer cell lines that had been treated with GDC-0449 (12-25 µM for 24 hours or 50 µM for 48 hours), GANT-61 (20 µM for 48 hours) or DMSO as described in the "Materials and methods". Given the great variability in the basal level of GLI2 expression, the mRNA expression levels were normalized to the GLI2 levels untreated SK-BR-3 cells, which displayed a relative expression level of approximately 2% of the expression level of the housekeeping gene HPRT. The scale for the GLI2 expression levels in MCF-10A cells is shown on the right. The results are expressed as the mean values of three independent experiments (* $p<0.05$ compared with the cultures treated with DMSO).

Supplementary Table 1: Effects of GDC-0449 (GDC) on cell cycle distribution in breast cancer cells.

			sub-G1[a]	p	G0/G1[a]	p	S[a]	p	G2/M[a]	p
**MCF-7**	DMSO		6.65		60.97		9.62		23.04	
	GDC (µM)	3	8.54	NS	62.03	NS	9.99	NS	19.79	NS
		6	6.53	NS	64.67	NS	10.12	NS	19.06	NS
		12	18.51	<0.001	59.49	NS	9.57	NS	13.39	<0.05
		25	22.76	<0.001	56.81	NS	7.75	<0.05	12.99	<0.05
**MDA-MB-468**	DMSO		6.28		50.00		9.36		34.77	
	GDC (µM)	3	7.01	NS	49.70	NS	9.88	NS	33.85	NS
		6	6.74	NS	52.00	NS	9.55	NS	32.10	NS
		12	6.96	NS	53.54	NS	9.83	NS	30.41	NS
		25	19.12	<0.001	5.92	<0.001	11.24	NS	64.10	<0.001
**MDA-MB-231***	DMSO		7.24		47.93		13.54		31.29	
	GDC (µM)	3	3.82	NS	48.55	NS	13.99	NS	33.55	NS
		6	3.75	NS	52.06	NS	14.90	NS	29.39	NS
		12	4.09	NS	52.28	NS	15.46	NS	28.34	NS
		25	3.50	NS	53.20	NS	14.57	NS	29.07	NS
**MDA-MB-453***	DMSO		9.07		49.63		12.87		28.92	
	GDC (µM)	3	7.92	NS	49.36	NS	11.25	NS	31.84	NS
		6	8.07	NS	52.52	NS	12.35	NS	27.59	NS
		12	8.66	NS	53.11	NS	10.13	NS	28.48	NS
		25	9.84	NS	60.01	NS	9.30	NS	21.12	NS
**T47-D**	DMSO		4.99		59.44		9.73		26.09	
	GDC (µM)	3	4.85	NS	57.82	NS	10.05	NS	27.28	NS
		6	5.65	NS	57.00	NS	9.90	NS	27.45	NS
		12	6.38	NS	53.99	<0.05	11.71	NS	28.25	NS
		25	13.99	<0.01	49.98	<0.01	12.02	NS	24.36	NS
**BT-474***	DMSO		9.93		67.86		7.07		15.34	

	GDC (µM)	3	8.53	NS	67.52	NS	7.30	NS	16.84	NS
		6	8.19	NS	69.10	NS	7.45	NS	15.59	NS
		12	9.57	NS	69.36	NS	6.67	NS	14.59	NS
		25	11.46	NS	67.38	NS	6.70	NS	14.64	NS
**SK-BR-3**	DMSO		6.60		55.53		5.15		33.06	
	GDC (µM)	3	7.57	<0.05	39.94	<0.05	5.10	NS	47.69	NS
		6	8.78	<0.01	41.26	<0.05	6.60	NS	44.68	NS
		12	10.72	<0.001	40.06	<0.05	8.06	NS	41.61	NS
		25	17.14	<0.001	35.39	<0.05	10.95	NS	37.20	NS
**TUBO***	DMSO		6.10		55.53		9.69		28.93	
	GDC (µM)	3	2.61	NS	57.36	NS	11.02	NS	29.31	NS
		6	3.83	NS	58.69	NS	10.00	NS	27.78	NS
		12	4.59	NS	59.70	NS	9.60	NS	26.35	NS
		25	6.31	NS	59.33	NS	9.87	NS	24.86	NS

[a] Percentage of cells in sub-G1, G0/G1, S and G2/M phases was calculated with CellQuest software. The results reported are mean values from three independent experiments.

*Cells treated for 96 hours. NS: not significant

Supplementary Table 2: Effects of GANT-61 (GANT) on cell cycle distribution in breast cancer cells.

			sub-G1[a]	p	G0/G1[a]	p	S[a]	p	G2/M[a]	p
**MCF-7**	DMSO		4.20		70.14		7.36		18.40	
	GANT (µM)	5	16.92	<0.01	63.47	<0.05	9.34	NS	10.51	<0.001
		10	62.03	<0.001	32.35	<0.001	2.84	<0.05	2.83	<0.001
		20	94.59	<0.001	3.73	<0.001	0.79	<0.01	0.90	<0.001
**MDA-MB-468**	DMSO		5.06		49.64		15.94		29.76	
	GANT (µM)	5	23.91	<0.001	46.92	<0.05	13.21	<0.05	16.38	<0.001
		10	84.12	<0.001	9.18	<0.001	3.05	<0.001	3.78	<0.001
		20	91.48	<0.001	5.07	<0.001	2.74	<0.001	0.85	<0.001
**MDA-MB-231**	DMSO		4.35		46.96		12.24		36.98	
	GANT (µM)	5	11.66	<0.05	50.13	NS	12.02	NS	26.59	<0.01
		10	37.62	<0.001	37.30	<0.001	10.74	NS	14.78	<0.001
		20	71.14	<0.001	14.12	<0.001	6.97	<0.05	8.15	<0.001
**MDA-MB-453**	DMSO		2.48		68.02		13.58		16.24	
	GANT (µM)	5	6.31	<0.05	68.21	NS	12.50	NS	13.27	<0.05
		10	62.95	<0.001	35.16	<0.001	1.58	<0.001	0.45	<0.001
		20	88.17	<0.001	9.25	<0.001	1.44	<0.001	1.25	<0.001
**T47-D**	DMSO		3.52		65.26		13.47		18.04	
	GANT (µM)	5	18.58	<0.05	54.86	<0.05	14.91	NS	12.05	<0.05
		10	37.14	<0.01	42.44	<0.01	9.65	<0.05	11.05	<0.05
		20	83.89	<0.001	11.40	<0.001	2.23	<0.01	2.53	<0.01
**BT-474**	DMSO		2.00		66.54		15.52		18.26	
	GANT (µM)	5	6.86	<0.05	68.41	NS	12.03	NS	12.29	<0.001
		10	39.33	<0.001	51.93	<0.001	4.62	<0.01	4.44	<0.001
		20	92.44	<0.001	5.50	<0.001	1.10	<0.01	1.38	<0.001
**SK-BR-3**	DMSO		6.41		54.86		9.99		29.09	

	GANT (µM)	5	11.52	<0.05	59.39	NS	8.09	NS	21.34	<0.01
		10	24.40	<0.001	63.62	NS	5.88	NS	6.57	<0.001
		20	67.37	<0.001	29.18	<0.001	1.47	<0.01	2.38	<0.001
**TUBO**	DMSO		8.98		43.60		6.86		40.96	
	GANT (µM)	5	6.38	NS	46.18	NS	7.91	NS	39.88	NS
		10	24.28	<0.01	41.95	NS	7.31	NS	26.84	<0.001
		20	96.59	<0.001	1.83	<0.001	0.15	<0.01	1.45	<0.001

[a] Percentage of cells in sub-G1, G0/G1, S and G2/M phases was calculated with CellQuest software. The results reported are mean values from three independent experiments. NS: not significant

Supplementary Table 3: Molecular features of employed breast cancer cell lines.

Cell line	ER*	PR	ErbB2	p53	Ras	Ref.
**T47-D**	+	+	+	+ M	+	[26-29]
**MCF-7**	+	+	+	+ WT	+	[26-29]
**SK-BR-3**	-	-	++	+ M	+	[26,28,29]
**MDA-MB-231**	-	-	+	+ M	+ M	[26-30]
**MDA-MB-468**	-	-	+	+ M	+	[26-30]
**BT-474**	+	+	++	+ M	+	[26-29]
**MDA-MB-453**	-	-	+/++	+ M	+ M	[26,27,29,31]
**MCF-10A**	-	-	+	+ WT	+	[26,28,30]

*Estrogen and progesterone receptors (ER/PR) status, ErbB2 expression, p53 and Ras protein levels and mutational status (M: mutated protein; WT: wild-type protein) are indicated.

Supplementary Table 4: Effects of GDC-0449 and GANT-61 on the expression of cleaved PARP-1, PARP-1, Bax/Bcl-2 ratio, p53, and on the expression and activation of ErbB receptors and pro-survival signaling pathway members in comparison to DMSO-treated cells.

Cell line	Treatment	Cleaved PARP-1	PARP-1	Bax/Bcl-2	p53	EGFR	ErbB2	pERK1/ ERK	pERK2/ ERK	p21-Ras	pAKT/ AKT
**T47-D**	**GDC-0449**	NO	=	↑ Bax*	↑	↓	=	=	=	n.a	n.a
**MCF-7**		NO	=	↑	↑	=	=	↓	↓	n.a	n.a
**SK-BR-3**		NO	↓	↑ Bax*	=	=	=	=	=	n.a	n.a
**T47-D**	**GANT-61**	NO	=	↑	↓	↓	↓	=	=	↓	=
**MCF-7**		YES	↓	↑	↑	↑	=	=	=	↓	n.d
**SK-BR-3**		NO	=	= Bax*	↓	↓	=	=	=	=	↑
**MDA-MB-231**		NO	=	=	↑	↓	n.d	↓	↓	↓	↓
**MDA-MB-468**		YES	=	=	=	↑	n.d	↓	↓	n.d	↓
**BT-474**		YES	=	=	=	↑	=	=	=	↓	↓
**MDA-MB-453**		NO	=	↑	↓	↓	↓	↓	↓	↓	n.d

= (equal); ↑ (increase); ↓ (decrease); *Bcl-2 was not detected.

n.a: not available; n.d: not detected
